# Substrate Induced Movement of the Metal Cofactor between Active and Resting State

**DOI:** 10.1002/anie.202213338

**Published:** 2022-11-09

**Authors:** Stefan R. Marsden, Hein J. Wijma, Michael K. F. Mohr, Inês Justo, Peter‐Leon Hagedoorn, Jesper Laustsen, Cy M. Jeffries, Dmitri Svergun, Luuk Mestrom, Duncan G. G. McMillan, Isabel Bento, Ulf Hanefeld

**Affiliations:** ^1^ Biokatalyse, Afdeling Biotechnologie Technische Universiteit Delft van der Maasweg 9 2629HZ Delft The Netherlands; ^2^ Groningen Biomolecular Sciences and Biotechnology Institute Faculty of Science and Engineering University of Groningen Nijenborg 4 9747AG Groningen The Netherlands; ^3^ EMBL Hamburg Notkestrasse 85 22607 Hamburg Germany

**Keywords:** Aldol Reaction, Class II Aldolase, Mn Metalloenzyme, Reaction Mechanism, Structure

## Abstract

Regulation of enzyme activity is vital for living organisms. In metalloenzymes, far‐reaching rearrangements of the protein scaffold are generally required to tune the metal cofactor's properties by allosteric regulation. Here structural analysis of hydroxyketoacid aldolase from *Sphingomonas wittichii* RW1 (*Sw*HKA) revealed a dynamic movement of the metal cofactor between two coordination spheres without protein scaffold rearrangements. In its resting state configuration (M^2+^_R_), the metal constitutes an integral part of the dimer interface within the overall hexameric assembly, but sterical constraints do not allow for substrate binding. Conversely, a second coordination sphere constitutes the catalytically active state (M^2+^_A_) at 2.4 Å distance. Bidentate coordination of a ketoacid substrate to M^2+^_A_ affords the overall lowest energy complex, which drives the transition from M^2+^_R_ to M^2+^_A_. While not described earlier, this type of regulation may be widespread and largely overlooked due to low occupancy of some of its states in protein crystal structures.

## Graphical abstract

Pyruvate dependent Class II aldolases require a ketoacid to induce a metal shift from resting state to active enzyme. This occurs without alterations in the protein structure and might be a common, but to date overlooked, feature in many metalloproteins.

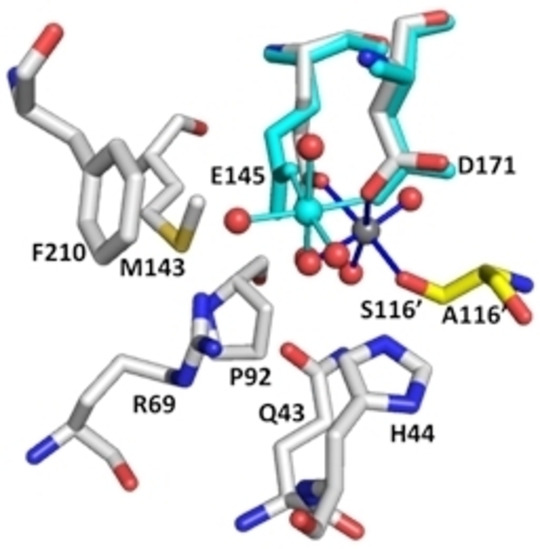

## Introduction

An estimated one third of all known enzymes require a metal cofactor to display their biological function.[^1^, ^2^, ^3^, ^4^] The formed protein‐metal complexes are typically well‐defined both in terms of coordination geometry, and their exact position within the respective metalloenzymes.[^5^, ^6^] In order to allow for a dynamic adaption to changed external conditions, the physicochemical properties of the metal center are commonly controlled by allosteric regulation.^[7]^ In this mechanism, far‐reaching rearrangements of the protein structure are generally required to tune the metal cofactor's properties via discrete movements of the coordinating residues.[^8^, ^9^, ^10^] Alternatively, a dynamic transition of the metal cofactor between distinct coordination sites could similarly alter its physicochemical properties without the need for a concomitant movement of protein residues. Although reports of this type are rare, such a dynamic behavior of the metal cofactor was previously identified as the key element in the catalytic mechanism of d‐xylose isomerase^[11]^ and in members of the medium chain alcohol dehydrogenase superfamily (MDR).[^12^, ^13^] In the MDR superfamily, zinc acts as a Lewis acid for the activation of aldehydes and ketones, which makes them susceptible to reduction by NAD(P)H.^[12]^ For the reaction to proceed in reverse, an increase in electron density is required to allow for the oxidation of alcohols by NAD(P)^+^. Depending on the direction of the reaction, the metal cofactor must therefore exert either an electron donating, or an electron withdrawing effect.^[12]^ This is achieved by variation between two coordination sites, which differently influence the protonation state of a coordinated water molecule and facilitate proton transfer in the required direction.^[12]^ Similarly, d‐xylose isomerase catalyzes the isomerization of d‐glucose to d‐fructose via the dynamic movement of one of its two metal cofactors. M1 remains stationary throughout the catalytic cycle and is responsible for substrate binding. Conversely, M2 alternates between two binding sites to facilitate the sequential activation of the C‐1 aldehyde (electron withdrawing effect for reduction at position M2a) and activation of the C‐2 hydroxyl group (electron donating effect for oxidation at position M2b) and *vice versa*. This dynamic transition between M2a and M2b facilitates the redox neutral hydride transfer for isomerization of the corresponding aldose and ketose forms.^[11]^ In both examples, the dynamic behavior of the metal cofactor constitutes an essential aspect of the enzymes’ catalytic mechanisms. However, other enzymatic functions are theoretically possible beyond the previously reported mechanistic applications.

Here, we describe the substrate induced movement of the metal cofactor between an inactive coordination sphere to a catalytically active one. This occurs for non‐mechanistic purposes within the divalent metal containing hydroxy ketoacid aldolase from *Sphingomonas wittichii* RW1 (*Sw*HKA). This enzyme displays an unusual promiscuity toward structural analogues of pyruvate,^[14]^ which were attributed to CH‐π interactions with aromatic amino acids that replaced a conserved leucine residue.^[15]^ Notably, *Sw*HKA is activated by trace amounts of inorganic phosphate (*K*_d,Pi_=175 μM), which effect a rate enhancement of up to two orders in magnitude for the first step of the aldol reaction, the deprotonation of the pyruvate.^[15]^ Its synthetic potential has been demonstrated for the conversion of various pyruvate analogues, including hydroxypyruvate,[^14^, ^15^] fluoropyruvate^[16]^ and 2‐oxobutyrate^[17]^ and derivatives.[^18^, ^19^, ^20^] Based on these reports, we conducted a detailed structural characterization of *Sw*HKA, which revealed two metal cofactor coordination sites of distinct properties. In contrast to allosteric regulation, the structural changes between the enzyme's active (M^2+^_A_) and resting state (M^2+^_R_) configurations are confined to the position of the metal cofactor. Most importantly, this transition seems to be induced by the binding of the pyruvate (‐derivate) as a ligand. These compounds selectively activate *Sw*HKA based on the characteristic structural motif of the ketoacid substrates.

## Results and Discussion

*Sw*HKA (accession number A5VH82, EC 4.1.2.52) was recombinantly expressed in *E. coli* BL21(DE3) and subsequently purified via its *N*‐terminal His_6_‐tag, followed by size‐exclusion chromatography to afford the *apo‐*enzyme in high purity, as described previously.^[15]^ The de novo crystal structure of wild‐type *apo‐Sw*HKA was solved by single‐wavelength anomalous dispersion of the sulfur atoms (S‐SAD), originating from two cysteine and six methionine residues per monomer (251 residues, PDBID 7O5I). This structure was subsequently used as a search model for solving the crystal structures of wild‐type and mutant variants of *Sw*HKA in the presence of different metals and substrates up to 1.2 Å resolution (Table S1).

**Figure 1:**
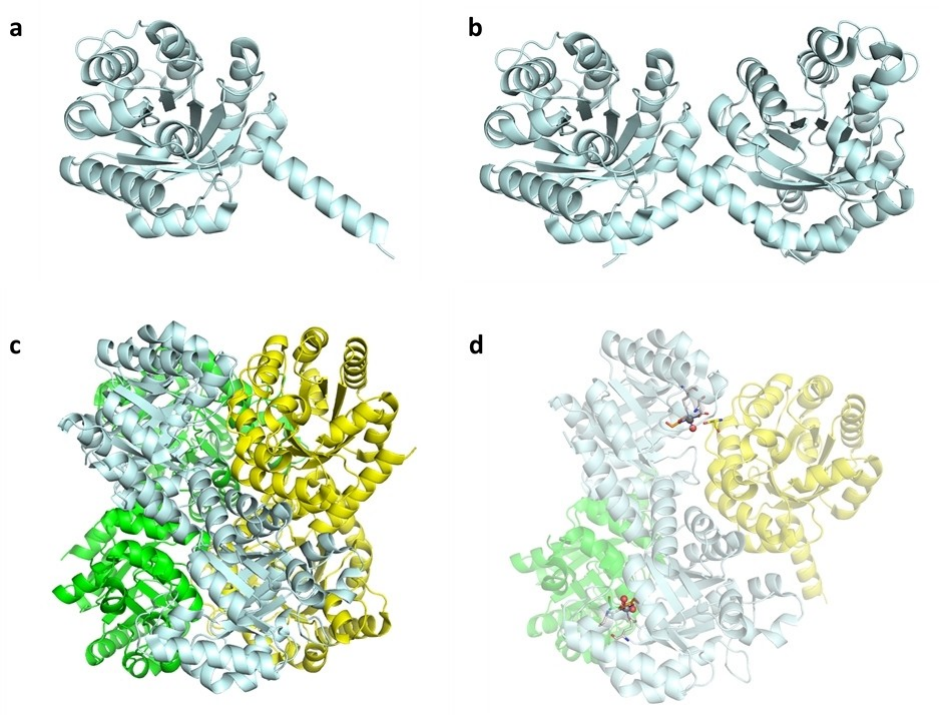
Crystal structure of *Sw*HKA (PDBID 6R62). a) TIM barrel fold monomeric *Sw*HKA. b) Two α‐helices pack onto β‐sheets to afford a dimer via domain swapping (both monomers in cyan). c) Trimerization of domain swapped dimers (green, yellow, cyan) affords the overall hexameric holoenzyme. d) Partial representation of *holo*‐*Sw*HKA, showing the location of active sites as ball and stick models at the interfaces of two domain‐swapped dimers. Each monomer participates in two different active sites. The second monomers (green, yellow) were omitted for visualization purposes. Figures were created with PyMOL.

*Holo*‐*Sw*HKA forms a trimer of homodimers, in which each monomer adopts an (α/β)_8_ triosephosphate isomerase (TIM) barrel fold (Figure 1). Domain swapping of the 8^th^ α‐helix packing onto a β‐sheet affords a dimer, and trimerization thereof generates the final hexameric assembly. In total, *holo*‐*Sw*HKA contains six equivalent active sites; each of which is formed from amino acids from two different domain‐swapped dimers. This makes *Sw*HKA a functional hexamer. Notably, the formation of the overall hexameric assembly of *apo‐Sw*HKA does not seem to require the presence of a metal cofactor.

**Figure 2:**
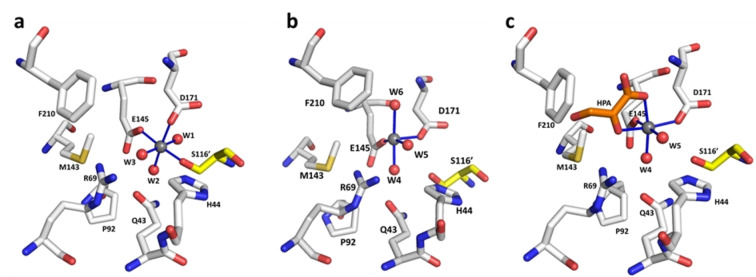
Resting and active state coordination spheres in *Sw*HKA. a) Octahedral M^2+^_R_ coordination sphere of the metal cofactor at the interface between two dimers (white; yellow, denoted by the prime symbol). M^2+^_R_ was observed in the following crystals structures: Holo‐*Sw*HKA‐Mg, Holo‐*Sw*HKA‐Mn, Holo‐F210W, Holo‐H44A—PDBID 7NUJ; 7O5R; 7O5W; 7O9R, respectively) b) Square pyramidal M^2+^_A,W_ coordination sphere with a vacant coordination site. This complex was observed with low occupancy in the absence of substrates in the crystal structure *Sw*HKA‐Mn (PDBID 7O5R). c) Coordination sphere of the catalytically active state M^2+^_A,S_. This complex was observed in the presence of substrate (hydroxypyruvate) in *Sw*HKA‐HPA, F201W‐HPA, S116A‐HKA (PDBID 6R62, 7O87 and 7NNK, respectively). The corresponding wall‐eye stereoview images are shown in Figure S3–S5. Figures were created with PyMOL.

Crystals of WT *apo*‐*Sw*HKA and mutant variants thereof were soaked with MgCl_2_ or MnCl_2_ to afford the *holo*‐enzyme. Under these conditions, the metal complex binds at the interface between two domain‐swapped dimers (Figure 1d, Figure 2a). Here, the metal is coordinated by residues E145 and D171 in a monodentate fashion, next to S116′ (where the prime symbol denotes residues from a second monomeric subunit), and three structural water molecules. In this configuration, replacement of either one of the three bound water molecules for substrate coordination is sterically impossible due to structural clashes with residues Q43, R69 and P92 (Figure 2a, Figure S1). Notably, the bidentate coordination of ketoacids was previously shown to be essential for catalytic activity.^[15]^ The coordination sphere S116′, E145 and D171, therefore, constitutes a catalytically inactive resting state of the enzyme (M^2+^_R_).

Conversely, a square pyramidal coordination sphere at 2.4 Å distance from M^2+^_R_ constitutes the catalytically active state of *holo*‐*Sw*HKA. Here, the coordination sphere no longer includes residue S116′, but only consists of E145, D171 next to three water molecules (M^2+^_A,W_, Figure 2b). The replacement of S116′ by a water ligand moves the metal cofactor toward the entrance of the active site, while the involved protein side chains retain their original position. Only M143 and E145 show a minor shift in the side chain position (approx. 1.0 Å) Figure S5b). This localized movement of the metal cofactor generates a vacant coordination site thereon. This newly generates sufficient space for bidentate substrate binding via the exchange of a single water ligand (W6; Figure 2c). In the absence of substrates, M^2+^_R_ constitutes the predominant state and the M^2+^_A,W_ complex was observed with lower occupancy in crystal structures of *holo*‐*Sw*HKA (Table S2). Assuming a dynamic equilibrium between both coordination spheres, M^2+^_R_ must therefore be lower in energy than M^2+^_A,W_.

In the presence of ketoacids, bidentate substrate coordination affords M^2+^_A,S_ as the lowest energy complex and predominant state in crystal structures. The M^2+^_A,S_ complex is additionally stabilized by side chain interactions from the protein environment with the coordinated substrate. In particular, CH‐π interactions between the electropositive C−H bonds at C‐3 in hydroxypyruvate (HPA) and the aromatic residue F210, play an important role.^[15]^ Bidentate substrate coordination therefore seems to constitute the driving force for the transition of the metal cofactor from M^2+^_R_ via M^2+^_A,W_ into the catalytically active configuration M^2+^_A,S_.

A stoichiometry of one metal ion per active site was determined by isothermal calorimetry titrations (ITC, Figure S23). Next to steric constraints, this observation excludes the simultaneous binding of two metal ions to both M^2+^_R_ and M^2+^_A_ in *Sw*HKA.

**Figure 3:**
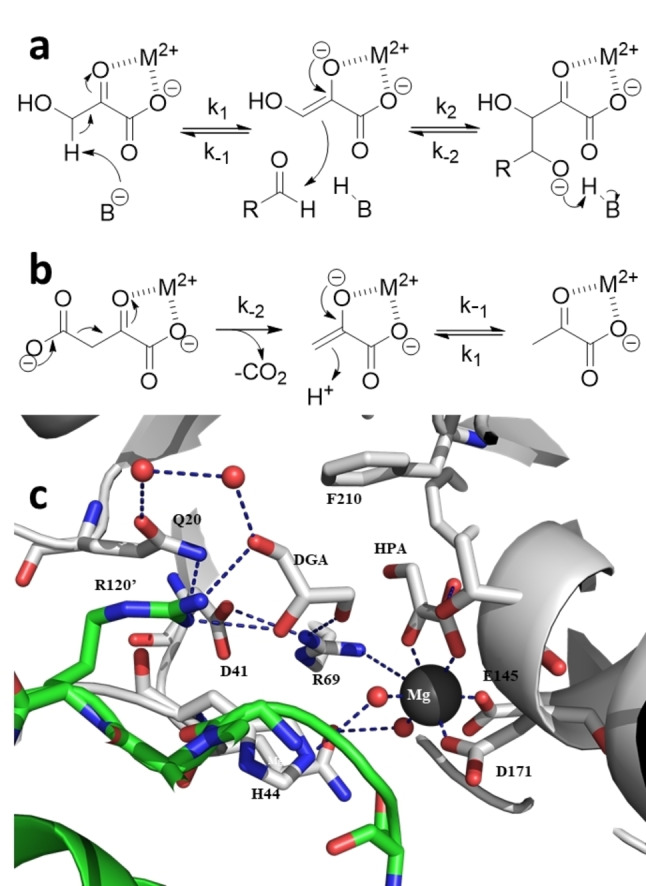
Reaction steps in *Sw*HKA catalysis. a) activity measurements via H/D exchange (k_1_). b) activity measurements via retro‐aldol reaction activity assay measuring k_‐2_ in the CO_2_ release. c) Close‐up view of the active site of *Sw*HKA with DGA bound (PDBID 8ADQ). Figure 3c was created with PyMOL.

To gain insight into the earlier observed activation of the enzyme by phosphate and a possible interaction thereof with the metal, phosphate was included into the crystallisation and soaking studies. Earlier reported class II pyruvate dependent aldolases (*Escherichia coli* DDG aldolase, PDBID 1DXE and *E. coli* Hpal aldolase, PDBID 4B5S) required phosphate for successful crystallisation and yielded phosphate containing crystals.[^21^, ^22^] Neither was the case for *Sw*HKA. Docking studies did however reveal a potential docking site, close to H44 (Figure S6a&b). An overlay with DDG aldolase and Hpal aldolase (Figure S6d) shows that the phosphate groups are very differently oriented in the different aldolases. The docking does however support the earlier proposed function in the pyruvate deprotonation, studied by H/D exchange (Figure 3a). It is also in line with results for a class II *Pseudomonas putida* aldolase (HMG/CHA aldolase).^[23]^ In both cases 100–300 fold improved deprotonation rates were observed. This could be enabled by a phosphate in concert with H44. The activity assay is based on the retro‐aldol type decarboxylation of oxaloacetate and displayed a significantly lower rate acceleration by phosphate (approx. 10 fold for both *Sw*HKA and HMG/CHA aldolase),[^15^, ^23^] and examines a different catalytic step (Figure 3b). Indeed, the oxaloacetate must bind partly in the position occupied by the phosphate in the docking.

With d‐glyceraldehyde (DGA) and HPA in the active site the position that phosphate occupied is fully taken (Figure 3c). The DGA is in a similar position as phosphate, interacting through hydrogen bonds with R69 and R120′ from the adjacent monomer. These interactions, and in particular the hydrogen bond to R69, orient the aldehyde for the nucleophilic attack by HPA (Figure 3c).

A structural alignment performed between the *Sw*HKA structure in complex with DGA (PDBID 8ADQ) and the crystal structures from *Acinetobacter baumannii* (*Ab*HpaI) in complex with (4*R*)‐2‐keto‐3‐deoxy‐d‐galactonate and with (4*S*)‐2‐keto‐3‐deoxy‐d‐gluconate (PDBID 7ETC and 7ETD, respectively),^[24]^ revealed a different configuration of the aldehyde orienting loop comprising residues 117′–121′ in *Sw*HKA (Figure S24). *Ab*HpaI has been shown to be highly selective for the 4*R*‐isomer,^[24]^ and indeed, this diastereoisomer is more tightly bound in the active site through hydrogen bonds to backbone nitrogen of A123′ and to the carbonyl oxygen of V120′ (Figure S24c). In the 4*S*‐isomer the aldehyde moiety only interacts indirectly with the protein chain through water molecules (Figure S24b). In *Sw*HKA the equivalent loop (residues 117′–121′) is shown to tightly interact with the aldehyde molecule through R120′ (Figure 3c and S24a). Whether these interactions are associated with *Sw*HKA stereoselectivity for the (3*S*,4*S*)‐isomer^[15]^ is unclear.

**Figure 4:**
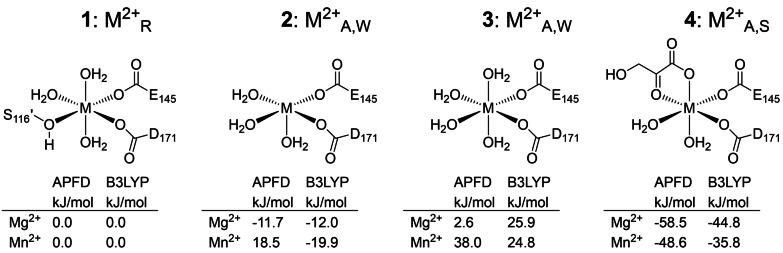
Metal complexes used for QM‐only calculations. Acetate and methanol ligands were used to approximate protein residues. Complex **1** represents M^2+^_R_, complex **2** represents M^2+^_A,W_. Complex **3** represents octahedral M^2+^_A,W_ (experimentally not observed), and complex **4** represents M^2+^_A,S_. Two different methods (APFD and B3LYP) were used for the calculation of relative energies of formation, which were normalized for the resting state M^2+^_R_. For complex **2**, only the calculations for high‐spin Mn^2+^ with APFD matched with experimental observations of M^2+^_R_ being lower in energy than square‐pyramidal M^2+^_A,W_.

Our findings suggest, that the three coordination states of the metal cofactor M^2+^_R_, M^2+^_A,W_ and M^2+^_A,S_ are part of a dynamic equilibrium, in which their relative distribution is determined by the energy difference between the corresponding metal complexes. We, therefore, set out to calculate the energies of formation using quantum mechanics (QM). The coordinating protein amino acids were approximated as acetate (for E145 and D171) and methanol (for S116′) molecules within an aqueous environment, which is common for a QM‐only approach.[^16^, ^25^, ^26^] While accurate predictions of the equilibrium distributions between M^2+^_R_, M^2+^_A,W_ and M^2+^_A,S_ would require extensive computational treatment of the whole active site environment, the general trend was in good agreement with experimental observations (Figure 4). The square‐pyramidal M^2+^_A,W_ complex was unanimously predicted for both Mg^2+^ and high‐spin Mn^2+^ in *Sw*HKA^[15]^ to be lower in energy than the corresponding hypothetical octahedral M^2+^_A,W_ complex with four water ligands. Thermodynamics, therefore, favor a square‐pyramidal coordination geometry for M^2+^_A,W_, which promotes substrate binding by providing a free coordination site. Similarly, M^2+^_A,S_ was unanimously predicted to constitute the overall lowest energy complex. This confirmed our previous notion, that bidentate substrate coordination constitutes the driving force for the enzyme's transition from the resting state into its catalytically active form.

In a previous study, several variants of *Sw*HKA were created at position F210 in order to probe the influence of aromatic electron density for CH‐π interactions with hydroxypyruvate.^[15]^ Here, the crystal structure of variant F210W soaked with MgCl_2_ and hydroxypyruvate could now be solved up to 1.2 Å resolution (PDBID 7OBU). While WT *Sw*HKA showed a monomer in the asymmetric unit with an occupancy of 1.0 for the metal cofactor in M^2+^_A,S_ and coordinated hydroxypyruvate (PDBID 6R62), variant F210W crystallized in a different space group, and a dimer was observed in the asymmetric unit (Figure S7). In this F210W crystal structure, monomer A exclusively contained the metal cofactor in the M^2+^_R_ configuration with an occupancy of 1.0, whereas monomer B displayed an occupancy of 0.7 for M^2+^_A,S_ and 0.3 for the catalytically inactive M^2+^_R_ (Table S2). Both monomers are structurally highly similar (rmsd=0.154 Å between 203 C atoms) and differ solely in some loops (Figure S8). Alignment of WT *Sw*HKA M^2+^_A,S_ with monomer B from variant F210W (containing M^2+^_A,S_/M^2+^_R_) showed no significant change in protein structure by the F210W mutation (Figure S9, rmsd=0.190 Å between 243 Cα atoms). Possible communication between subunits in the form of allosteric regulation could not be identified, as the two active sites were equivalent.

*Sw*HKA displays activity both with Mn^2+^ and Mg^2+^. Soaking of WT *apo*‐*Sw*HKA crystals with either Mg^2+^ or Mn^2+^ in the absence of substrates did not result in any change of protein structure (PDBID 7NUJ, 7O5R, for Mg^2+^ and Mn^2+^, respectively; Figure S10, rmsd=0.195 Å between 210 Cα atoms), but led to different distributions between M^2+^_R_ and M^2+^_A,W_. With Mg^2+^, the M^2+^_R_ configuration showed occupancy of 1.0, whereas a mixture of M^2+^_R_ (0.61) and M^2+^_A,W_ (0.36) was observed for Mn^2+^. In this case, the complete saturation of both active sites with the metal cofactors suggests that the observed relative occupancy of each state accurately reflects its actual equilibrium distribution. The energy difference between M^2+^_R_ and M^2+^_A,W_ in *Sw*HKA therefore, seems to be substantially larger for protein‐metal complexes with Mg^2+^ than with Mn^2+^.

**Figure 5:**
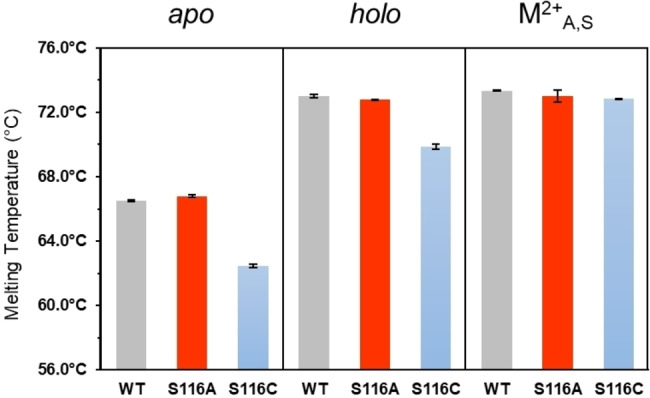
Protein melting temperatures. Thermal unfolding was investigated by nano‐differential scanning fluorimetry for variants of *Sw*HKA under different conditions. The holoenzyme state could correspond to both M^2+^_R_ and M^2+^_A,W_. Conditions: *Sw*HKA (5 mg mL^−1^), MgCl_2_ (10 mM), hydroxypyruvate (20 mM), in Hepes buffer (20 mM, pH 7.5).

To investigate a potential structural role for the metal cofactor in the M^2+^_R_ configuration through interactions with residue S116′, two different variants were designed at this position. Notably, mutagenesis at position S116 reduced the expression yield of soluble protein by more than 10‐fold down to ≈3 mg L^−1^ of expression medium. In the first variant, serine was replaced by alanine (S116A) in order to circumvent the formation of M^2+^_R_. Indeed, the metal cofactor was found in the M^2+^_A,W_ configuration (Figure S12, S116A PDBID 7NR1). Identical orientations of the alanine/serine residues were observed, and the protein fold was unaffected by the mutation (rmsd=0.115 Å between 230 Cα atoms). However, the substrate bound complex M^2+^_A,S_ showed a strangely distorted geometry (Figure S13, S116A‐HPA PDBID 7NNK), which could explain the observed difference in the catalytic activity of this variant (Table 1). Conversely, a cysteine mutation (S116C) was introduced to potentially alter the binding in M^2+^_R_. Unfortunately, no well‐diffracting crystals could be obtained for the S116C variant despite continued efforts, which limited our analysis of this variant to the interpretation of biochemical data. Being an integral part of the dimer interface, M^2+^_R_ was expected to increase the thermal stability of wild‐type holo‐*Sw*HKA. We, therefore, determined protein melting temperatures using nano‐differential scanning fluorimetry (nanoDSF, Figure 5). In the absence of metals, wild‐type *apo*‐*Sw*HKA and *apo*‐S116A displayed similar melting temperatures (*T*_m_) of approximately 66 °C, while variant S116C showed a slightly lower stability of 62 °C, which suggests no substantial disruption of the protein fold. Surprisingly, no significant difference was observed between the wild‐type and S116 A variants of *Sw*HKA upon holoenzyme formation with MgCl_2_, as the thermal stability of all variants increased by ≈7 °C. This suggests that the thermal unfolding of *Sw*HKA is not initiated by the dissociation of the trimer of the domain‐swapped dimers. This prevents measurements of the potential increase in stability by the formation of M^2+^_R_.

**Table 1:** Overview of metal dissociation constants (*K*_d_) and maximum rates (*v*_sat_) for *Sw*HKA variants under different conditions. Assays were carried out in the presence and absence of inorganic phosphate as kinetic activator, based on the retro‐aldol type cleavage of oxaloacetate into pyruvate.^[15]^ Since activity was used as reporter, the data shown are with respect to the M^2+^_A,S_ complex.

Variant	M^2+^	Phosphate	*v*_sat_ [U mg^−1^]	*K*_d_ [μM]
WT^[15]^	Mg^2+^	–	1.65±0.03	58.4±4.1
S116A	Mg^2+^	–	3.66±0.05	37.9±3.0
S116C	Mg^2+^	–	2.78±0.12	80.4±19.4
				
WT^[15]^	Mg^2+^	+	17.21±0.29	158±12
S116A	Mg^2+^	+	9.27±0.27	105±18
S116C	Mg^2+^	+	5.23±0.1	179±16
				
WT^[15]^	Mn^2+^	–	2.15±0.09	3.3±0.6
S116A	Mn^2+^	–	3.2±0.09	3.4±0.6
S116C	Mn^2+^	–	2.3±0.06	4.1±0.6
				
WT^[15]^	Mn^2+^	+	27.0±0.67	6.5±0.7
S116A	Mn^2+^	+	8.72±0.22	9.1±1.3
S116C	Mn^2+^	+	4.28±0.16	26.0±5.2

In the presence of hydroxypyruvate, all three variants displayed the same melting temperature of 73 °C. This is in line with expectations, as the M^2+^_A,S_ complex is estimated to be largely identical in all three variants.

The Mn^2+^ cofactor in WT *Sw*HKA was previously analyzed by electron paramagnetic resonance (EPR), and showed an unusually broad signal^[15]^ due to a distorted octahedral geometry.^[27]^ Analysis of the Mn^2+^ complexes of *holo*‐*Sw*HKA variants S116A and S116C in the absence of hydroxypyruvate showed largely identical EPR signals (Figure S22a). The cysteine residue in S116C, therefore, does not seem to be involved in the coordination of the metal cofactor in M^2+^_R_. In the presence of hydroxypyruvate, both variants similarly showed the previously reported broad EPR signal as a result of substrate coordination (Figure S22b).

The results of SEC‐SAXS‐MALLS analyses (Figure S11 and Table S3) demonstrate that the predominant assembly of WT *Sw*HKA and the corresponding S116A and S116C mutants are globular hexamers. The conformation adopted in solution is very similar to the symmetry‐related trimer‐of‐dimers that are observed within the extended crystal lattice. The spatial disposition of the hexamers is not significantly affected by the mutations, and the X‐ray crystal structure of the hexamer fits the solution scattering data well (*χ* ^2^ 1.11–1.14, CorMap P; 0.001–0.09).

The affinity of the different enzyme variants towards Mg^2+^ and Mn^2+^ as cofactors was investigated both in the presence and absence of phosphate as a kinetic activator for *Sw*HKA.^[15]^ For this purpose, retro‐aldol activity was used as a reporter to determine the apparent constants *K*_d_ and *v*_sat_ for the M^2+^_A,S_ complex in metal saturation experiments (Table 1, Figure S14–S21). For the S116A mutant, a lower apparent *K*_d_ (i.e. higher affinity) toward M^2+^_A,S_ was observed with Mg^2+^, while the affinity remained mostly unaffected when Mn^2+^ was used. These data suggest that elimination of M^2+^_R_ could remove the competition between the two binding modes, and thereby increase its affinity toward the M^2+^_A,S_ configuration. Slightly higher apparent metal dissociation constants (i.e. lower affinity toward M^2+^_A,S_) were observed with variant S116C. This could be due to unfavorable sterical effects, which are difficult to rationalize in the absence of a crystal structure, but would be in line with its lower melting temperature (Figure 5). The dissociation constant of *Sw*HKA for Mn^2+^ was 37.6 μM measured by ITC in the absence of the substrate oxaloacetate, while a *K*_d_ of 3.3±0.6 μM was reported previously for Mn^2+^ binding *Sw*HKA in equilibrium binding titrations using activity as a reporter (Table 1). We attribute this difference to the effect of oxaloacetate, which binds to the metal, on the metal affinity of the enzyme.

Our findings differ from previously published reports on members of the MDR superfamily by the electronic‐, geometric‐ and functional characteristics of the metal centers. Zn^2+^ has an [Ar] 3d^10^ electron configuration and preferentially adopts a tetrahedral coordination geometry, next to the transient formation of trigonal‐bipyramidal complexes during ligand exchange.^[28]^ In contrast, Mg^2+^ is completely devoid of d‐electrons ([Ne] 3s^0^), while high‐spin Mn^2+^ only contains five half‐filled d‐orbitals ([Ar] 3d^5^). Both metals generally favor the formation of octahedral complexes, with the additional observation of square‐pyramidal complexes in *Sw*HKA. In MDRs and d‐xylose isomerase, the dynamic properties of the metal cofactors constitute a key element of their respective catalytic mechanisms, which are required for the reactions to be reversible. In contrast, the movement between M^2+^_R_ and M^2+^_A,S_ does not serve a mechanistic purpose in *Sw*HKA, but corresponds to a binary switch between two states of distinct properties. No indications that phosphate influences this process were detected.

In the case of another TIM barrel Class II aldolase, fructose 1,6‐ biphosphate aldolase (FBA), a shift of the metal was also observed.^[29]^ The catalytic Zn^2+^ was either observed in a metal keep position and a catalytic active position. In the metal keeping position the Zn^2+^ is tetrahedral coordinated and migrates almost 4 Å into a surface exposed catalytic site. In the presence of di‐hydroxy‐acetone phosphate (DHAP) Zn^2+^ is suggested to enable enolate formation. After the carbon‐carbon bond formation has taken place Zn^2+^ is found in the metal keeping position, even when the product is coordinated.^[30]^

While the natural function of the *Sw*HKA M^2+^_R_ configuration could not yet be determined, an environment responsive regulation between catalytic activity and enhanced stability is conceptually plausible for this mechanism. This could be similar to what was observed for FBA, however, it needs to be emphasized that the active sites are in very different locations of the common TIM barrel.

Additionally, the requirement for bidentate substrate coordination to transform *Sw*HKA into its catalytically active state constitutes a powerful prerequisite, that increases its substrate specificity. This mechanism of control could find potential applications in the design of artificial metalloenzymes. Here, the bio‐compatibility of the (organo‐)metallic catalyst is considerably improved by the protein environment, which then allows for its modular incorporation as part of multi‐step enzymatic cascade reactions.[^31^, ^32^, ^33^, ^34^, ^35^, ^36^, ^37^] Similar to the regulation of metabolism in vivo, control of enzyme activity and selectivity becomes of great importance with increasing complexity of the synthetic cascade reaction. The substrate induced transition from a resting‐ into a catalytically active‐state could therefore increase their substrate specificity and constitute an interesting alternative to the design of allosteric regulation in artificial metalloenzymes.^[10]^

## Conclusion

The structural characterization of *Sw*HKA revealed a movement of its metal cofactor between two coordination sites of distinct properties, which are interconnected by a dynamic equilibrium. The transition from M^2+^_R_ to M^2+^_A,S_ is induced by bidentate substrate binding and occurs without a concomitant rearrangement of the protein scaffold. The equilibrium distribution between the individual states is determined by their relative energy difference, which can be modulated both via reversible ligand exchange and the use of different metals. This mechanism, therefore, constitutes an alternative to allosteric regulation for the adjustment of metalloenzyme properties in response to a changed external environment. The conceptual simplicity of a dynamic equilibrium between different metal binding sites suggests, that this behavior may be a more widespread phenomenon in nature, which has been largely overlooked until now due to its conditional character and the low occupancy of some of its states in protein crystal structures.

## Conflict of interest

The authors declare no conflict of interest.

1

## Supporting information

As a service to our authors and readers, this journal provides supporting information supplied by the authors. Such materials are peer reviewed and may be re‐organized for online delivery, but are not copy‐edited or typeset. Technical support issues arising from supporting information (other than missing files) should be addressed to the authors.

Supporting Information

## Data Availability

The data that support the findings of this study are available from the corresponding author upon reasonable request.
