# Supplementary material for: Substrate Induced Movement of the Metal Cofactor between Active and Resting State
Source: Angew Chem Int Ed Engl. 2022 Nov 9;61(49):e202213338. doi: 10.1002/anie.202213338 (PMC10099721; doi:10.1002/anie.202213338)
Supplement: Supplementary file 1 — Supporting Information [file ANIE-61-0-s001.pdf]

Angewandte Chemie
Eine Zeitschrift der Gesellschaft Deutscher Chemiker

Supporting Information

Substrate Induced Movement of the Metal Cofactor between Active and Resting State

S. R. Marsden, H. J. Wijma, M. K. F. Mohr, I. Justo, P.-L. Hagedoorn, J. Laustsen, C. M. Jeffries, D. Svergun, L. Mestrom, D. G. G. McMillan, I. Bento, U. Hanefeld**

Supplementary Information for

This PDF file includes:

Supplementary text

Figures S1 to S24

Tables S1 to S4

SI References

Supplementary Information

Materials **3**

Gene and protein sequences **4**

Expression and purification **9**

Determination of metal dissociation constants *via* activity assays **11**

Surface and stereoview of the different metal complexes in *Sw*HKA **13**

Alignment of different variants and states of *Sw*HKA monomers/dimers **17**

SEC-SAXS MALLS **22**

Metal dissociation constants and kinetic curves **26**

EPR spectrums of S116A and S116C **30**

ITC titration of WT *Sw*HKA with $MgCl_2$ **33**

X-ray Crystallography **35**

Phosphate Docking **36**

SAXS **37**

Nano-Differential scanning fluorimetry (nanoDSF) **40**

Refinement statistics **41**

QM energy calculations. **42**

Supplementary References **43**

Materials

Chemicals were generally bought in the highest purity that was commercially available and were used without further purification, unless stated otherwise. Microbiological work was carried out under sterile conditions using autoclaved materials and solutions. Potassium dihydrogen phosphate (Sigma Aldrich, 99.5%), dipotassium hydrogen phosphate (Sigma Aldrich, ≥ 98%), magnesium chloride hexahydrate (J.T. Baker), sodium chloride (Sigma Aldrich, ≥99.5%), triethanolamine hydrochloride (Sigma Aldrich, 99.5%), imidazole (Sigma Aldrich, ≥99%), Ni-sepharose® 6Fast Flow (GE Healthcare, 6% cross-linked agarose), P-2 Bio-Gel (100 - 1800 Da, Bio-Rad), potassium hydroxide (Acros Organics, 85%), L-lactic dehydrogenase type II from rabbit muscle (Sigma Aldrich, ammonium sulfate suspension, 800-1200 U/mg, EC 1.1.1.27), yeast extract (BD Becton Dickinson), tryptone (BD Bacto™ Peptone, BD Biosciences), IPTG (Thermo Fisher, dioxane free), ampicillin sodium salt (Sigma Aldrich), lysozyme (Sigma Aldrich, from chicken egg white, lyophilized powder, ≥90%, ≥40000 U/mg, EC 3.2.1.17), deoxyribonuclease I (Sigma Aldrich, from bovine pancreas, lyophilized powder, ≥85%, ≥400 Kunitz U/mg, EC 3.1.21.1), cOmplete EDTA-free protease inhibitor cocktail (Sigma Aldrich), NADH (Prozomix, UK), Pierce BCA protein assay kit (Thermo Fisher Scientific). VP-ITC MicroCal, (Malverne).

Gene and protein sequences of the hydroxy ketoacid aldolase from *Sphingomonas wittichii* RW1 (*Sw*HKA), plasmid pSWIT02, accession number A5VH82:[1]

Protein sequence **wild-type**:

MHHHHHHNKVRTCWNEGRPALAGWLQLPGTLHAEALARLDYDAVVIDMQHSPIDFGQVAPML

IAIELGGAEPFVRTQVNDPSDIMKLLDAGAYGIIAPMVNTRAEAQTLASALHYSPRGLRSFG

PRRPSLRYGSGYLAQASETVVGLAMIETREALANIDEILSVDGIDGVFIGPTDLALDLGHAP

LVDTEEAEVVSAIAHVRERAHAAGKRVGIFCGSGGFARVKLAEGFDFVTAAPDLAMLSAAAR

QVIADARAL

Protein sequence *Sw*HKA **S116A**:

MHHHHHHNKVRTCWNEGRPALAGWLQLPGTLHAEALARLDYDAVVIDMQHSPIDFGQVAPML

IAIELGGAEPFVRTQVNDPSDIMKLLDAGAYGIIAPMVNTRAEAQTLASALHYSPRGLR**A**FG

PRRPSLRYGSGYLAQASETVVGLAMIETREALANIDEILSVDGIDGVFIGPTDLALDLGHAP

LVDTEEAEVVSAIAHVRERAHAAGKRVGIFCGSGGFARVKLAEGFDFVTAAPDLAMLSAAAR

QVIADARAL

Protein sequence *Sw*HKA **S116C**:

MHHHHHHNKVRTCWNEGRPALAGWLQLPGTLHAEALARLDYDAVVIDMQHSPIDFGQVAPML

IAIELGGAEPFVRTQVNDPSDIMKLLDAGAYGIIAPMVNTRAEAQTLASALHYSPRGLR**C**FG

PRRPSLRYGSGYLAQASETVVGLAMIETREALANIDEILSVDGIDGVFIGPTDLALDLGHAP

LVDTEEAEVVSAIAHVRERAHAAGKRVGIFCGSGGFARVKLAEGFDFVTAAPDLAMLSAAAR

QVIADARAL

Protein sequence *Sw*HKA **F210W**:[1]

MHHHHHHNKVRTCWNEGRPALAGWLQLPGTLHAEALARLDYDAVVIDMQHSPIDFGQVAPML

IAIELGGAEPFVRTQVNDPSDIMKLLDAGAYGIIAPMVNTRAEAQTLASALHYSPRGLRSFG

PRRPSLRYGSGYLAQASETVVGLAMIETREALANIDEILSVDGIDGVFIGPTDLALDLGHAP

LVDTEEAEVVSAIAHVRERAHAAGKRVGI**W**CGSGGFARVKLAEGFDFVTAAPDLAMLSAAAR

QVIADARAL

Native gene sequence of WT *Sw*HKA from *Sphingomonas wittichii* RW1 pSWIT02, accession number A5VH82:

ATGAATAAGGTACGAACATGCTGGAACGAGGGGCGGCCCGCGCTCGCGGGGTGGCTGCAACT

GCCCGGAACCCTCCACGCCGAGGCGCTCGCGCGGCTCGATTATGACGCAGTCGTCATCGATA

TGCAGCATAGCCCGATCGATTTCGGTCAGGTTGCGCCGATGCTGATAGCGATCGAGCTAGGC

GGCGCCGAACCGTTCGTGCGCACGCAAGTCAACGATCCGTCGGATATCATGAAACTGCTCGA

TGCGGGCGCCTATGGCATAATCGCGCCGATGGTGAACACCCGCGCGGAAGCGCAAACATTGG

CGTCTGCCCTGCATTATTCGCCGCGCGGTCTCCGCTCCTTCGGTCCGCGACGGCCATCCCTG

CGGTACGGCTCGGGCTATCTCGCGCAGGCGAGCGAGACCGTGGTCGGGCTGGCGATGATCGA

GACCCGCGAAGCGCTGGCGAATATCGACGAGATCCTGTCGGTAGACGGGATCGACGGCGTAT

TCATCGGTCCGACCGATCTCGCGCTCGATCTGGGCCATGCCCCGCTGGTCGATACCGAGGAG

GCGGAGGTGGTATCCGCCATCGCGCATGTGCGGGAACGTGCTCATGCGGCCGGCAAGCGGGT

CGGCATCTTCTGCGGCAGCGGCGGCTTTGCGCGGGTCAAGCTGGCTGAAGGCTTCGATTTCG

TCACCGCCGCCCCCGACCTTGCCATGCTGAGTGCTGCCGCCCGCCAGGTCATCGCGGACGCC

AGGGCGCTTTGA

Codon optimized gene sequence of WT *Sw*HKA for heterologous expression in *E. coli* BL21(DE3):

ATGCATCACCACCACCATCATAATAAAGTTCGTACCTGCTGGAACGAAGGTCGTCCGGCGTT

AGCAGGTTGGCTGCAGCTGCCAGGGACCCTGCACGCAGAAGCGCTGGCTCGTCTTGATTATG

ATGCAGTTGTGATCGATATGCAGCACAGCCCGATCGACTTCGGCCAGGTTGCGCCGATGCTG

ATCGCGATCGAACTGGGTGGTGCGGAACCGTTCGTTCGTACCCAGGTTAACGATCCGTCTGA

CATCATGAAACTGCTGGATGCTGGCGCATACGGTATCATTGCACCGATGGTAAATACCCGCG

CTGAAGCACAGACCCTGGCATCTGCGCTGCATTATAGCCCGCGTGGTCTGCGTTCTTTTGGT

CCGCGTCGTCCGTCTTTGCGCTATGGTAGCGGTTATCTGGCTCAGGCATCTGAAACCGTTGT

TGGTCTGGCAATGATTGAAACTCGTGAAGCACTGGCTAACATTGATGAAATTTTAAGCGTTG

ATGGTATTGATGGTGTTTTTATTGGTCCGACTGATTTAGCTCTGGATCTTGGTCACGCGCCG

TTAGTAGATACCGAAGAAGCTGAAGTAGTTTCTGCAATTGCCCACGTTCGTGAACGTGCACA

CGCTGCTGGTAAACGTGTTGGTATCTTTTGCGGCTCTGGTGGTTTCGCGCGTGTTAAACTGG

CTGAAGGTTTTGATTTCGTTACTGCGGCTCCGGATCTGGCAATGCTGTCTGCTGCAGCTCGT

CAGGTTATTGCTGATGCGCGTGCGCTGTAA

Codon optimized gene sequence of *Sw*HKA S116A for heterologous expression in *E. coli* BL21(DE3). The construct was synthesized by BaseClear B.V.

ATGCATCACCACCACCATCATAATAAAGTTCGTACCTGCTGGAACGAAGGTCGTCCGGCGTT

AGCAGGTTGGCTGCAGCTGCCAGGGACCCTGCACGCAGAAGCGCTGGCTCGTCTTGATTATG

ATGCAGTTGTGATCGATATGCAGCACAGCCCGATCGACTTCGGCCAGGTTGCGCCGATGCTG

ATCGCGATCGAACTGGGTGGTGCGGAACCGTTCGTTCGTACCCAGGTTAACGATCCGTCTGA

CATCATGAAACTGCTGGATGCTGGCGCATACGGTATCATTGCACCGATGGTAAATACCCGCG

CTGAAGCACAGACCCTGGCATCTGCGCTGCATTATAGCCCGCGTGGTCTGCGT**GCG**TTTGGT

CCGCGTCGTCCGTCTTTGCGCTATGGTAGCGGTTATCTGGCTCAGGCATCTGAAACCGTTGT

TGGTCTGGCAATGATTGAAACTCGTGAAGCACTGGCTAACATTGATGAAATTTTAAGCGTTG
ATGGTATTGATGGTGTTTTTATTGGTCCGACTGATTTAGCTCTGGATCTTGGTCACGCGCCG
TTAGTAGATACCGAAGAAGCTGAAGTAGTTTCTGCAATTGCCCACGTTCGTGAACGTGCACA
CGCTGCTGGTAAACGTGTTGGTATCTTTTGCGGCTCTGGTGGTTTCGCGCGTGTTAAACTGG
CTGAAGGTTTTGATTTCGTTACTGCGGCTCCGGATCTGGCAATGCTGTCTGCTGCAGCTCGT
CAGGTTATTGCTGATGCGCGTGCGCTGTAA

Codon optimized gene sequence of *Sw*HKA S116C for heterologous expression in *E. coli* BL21(DE3). The construct was synthesized by BaseClear B.V.

ATGCATCACCACCACCATCATAATAAAGTTCGTACCTGCTGGAACGAAGGTCGTCCGGCGTT
AGCAGGTTGGCTGCAGCTGCCAGGGACCCTGCACGCAGAAGCGCTGGCTCGTCTTGATTATG
ATGCAGTTGTGATCGATATGCAGCACAGCCCGATCGACTTCGGCCAGGTTGCGCCGATGCTG
ATCGCGATCGAACTGGGTGGTGCGGAACCGTTCGTTCGTACCCAGGTTAACGATCCGTCTGA
CATCATGAAACTGCTGGATGCTGGCGCATACGGTATCATTGCACCGATGGTAAATACCCGCG
CTGAAGCACAGACCCTGGCATCTGCGCTGCATTATAGCCCGCGTGGTCTGCGT**TGC**TTTGGT
CCGCGTCGTCCGTCTTTGCGCTATGGTAGCGGTTATCTGGCTCAGGCATCTGAAACCGTTGT
TGGTCTGGCAATGATTGAAACTCGTGAAGCACTGGCTAACATTGATGAAATTTTAAGCGTTG
ATGGTATTGATGGTGTTTTTATTGGTCCGACTGATTTAGCTCTGGATCTTGGTCACGCGCCG
TTAGTAGATACCGAAGAAGCTGAAGTAGTTTCTGCAATTGCCCACGTTCGTGAACGTGCACA
CGCTGCTGGTAAACGTGTTGGTATCTTTTGCGGCTCTGGTGGTTTCGCGCGTGTTAAACTGG
CTGAAGGTTTTGATTTCGTTACTGCGGCTCCGGATCTGGCAATGCTGTCTGCTGCAGCTCGT
CAGGTTATTGCTGATGCGCGTGCGCTGTAA

Codon optimized gene sequence of *Sw*HKA F210W for heterologous expression in *E. coli* BL21(DE3):[1]

ATGCATCACCACCACCATCATAATAAAGTTCGTACCTGCTGGAACGAAGGTCGTCCGGCGTT

AGCAGGTTGGCTGCAGCTGCCAGGGACCCTGCACGCAGAAGCGCTGGCTCGTCTTGATTATG

ATGCAGTTGTGATCGATATGCAGCACAGCCCGATCGACTTCGGCCAGGTTGCGCCGATGCTG

ATCGCGATCGAACTGGGTGGTGCGGAACCGTTCGTTCGTACCCAGGTTAACGATCCGTCTGA

CATCATGAAACTGCTGGATGCTGGCGCATACGGTATCATTGCACCGATGGTAAATACCCGCG

CTGAAGCACAGACCCTGGCATCTGCGCTGCATTATAGCCCGCGTGGTCTGCGTTCTTTTGGT

CCGCGTCGTCCGTCTTTGCGCTATGGTAGCGGTTATCTGGCTCAGGCATCTGAAACCGTTGT

TGGTCTGGCAATGATTGAAACTCGTGAAGCACTGGCTAACATTGATGAAATTTTAAGCGTTG

ATGGTATTGATGGTGTTTTTATTGGTCCGACTGATTTAGCTCTGGATCTTGGTCACGCGCCG

TTAGTAGATACCGAAGAAGCTGAAGTAGTTTCTGCAATTGCCCACGTTCGTGAACGTGCACA

CGCTGCTGGTAAACGTGTTGGTATC**TGG**TGCGGCTCTGGTGGTTTCGCGCGTGTTAAACTGG

CTGAAGGTTTTGATTTCGTTACTGCGGCTCCGGATCTGGCAATGCTGTCTGCTGCAGCTCGT

CAGGTTATTGCTGATGCGCGTGCGCTGTAA

Expression of WT *Sw*HKA and mutant variants:[1]

The genes coding for the *Sw*HKA wild-type and mutant variants were synthesized by BaseClear B.V. (The Netherlands) in the pET22b(+) plasmid and chemically competent *E. coli* BL21(DE3) cells were transformed with the constructs using the following protocol:

Transformation: Ca^{2+} competent *E. coli* BL21(DE3) cells (50 µL) were thawed on ice (30 minutes), after which they were incubated with plasmid solution (2 µL, 4 ng/µL, 30 minutes, on ice). The cells were exposed to a heat shock (45 seconds, 42°C), followed by incubation on ice (2 minutes). The cells were then allowed to warm up to room temperature before the gentle addition of LB medium (500 µL), followed by incubation (1 hour, 37°C). From this solution, 50 µL and 200 µL were plated on selective agar plates (100 µg/mL ampicillin) and incubated overnight (37°C).

Preparation of TB medium: Tryptone (12 g/L), yeast extract (24 g/L), and glycerol (8 mL/L) were dissolved in milliQ water (900 mL) and the solution was autoclaved at 121°C. K_2HPO_4 (9.4 g/L), KH_2PO_4 (2.2 g/L) were dissolved in milliQ (100 mL) and autoclaved separately. Both solutions were combined upon sterilization to make up 1 liter of TB medium, pH 7.2.

Expression: Pre-cultures were grown overnight from single colonies (200 mL LB medium in a 1 L Erlenmeyer flask, 100 µg/mL ampicillin, 1% w/v glucose, 37°C, 180 rpm) and were used as inoculum (1:50 dilution, 1 L TB medium in a baffled 5 L Erlenmeyer flask, 37°C, 140 rpm, containing 100 µg/mL ampicillin and 1% w/v glucose). Expression was induced at OD_{600} = 0.6-0.8 by the addition of filter sterilized isopropyl β-D-1-thiogalactopyranoside (IPTG, 400 µM final concentration) and expression was carried out for 16 hours at reduced temperature (25°C, 140 rpm). The cells were harvested by centrifugation (17,000 × *g*, 10 minutes, 4°C) and the pellet was stored at -80°C.

Affinity purification:

Buffer A: 20 mM imidazole, 500 mM NaCl, 20 mM TEOA, pH 7.4

Buffer B: 500 mM imidazole, 500 mM NaCl, 20 mM TEOA, pH 7.4

Lysis buffer: Buffer A + lysozyme (2 mg/mL) a spatula tip of deoxyribonuclease I, cOmplete Protease Inhibitor Cocktail (1 tablet/10 mL).

The pellet was homogenized with lysis buffer (2 mL/g wet cell weight) and the cells were disrupted with a Constant SystemsTM cell disrupter (three cycles, 1.4 kbar). The debris was pelleted by centrifugation (17,000 × *g*, 30 minutes) and the supernatant was filtered (0.45 µm pore size) before it was loaded onto a custom packed column (XK16/20 column) containing Ni-sepharose 6 fast-flow resin (15 mL, GE Life Sciences) using a NGC chromatography system (3 mL/min, Bio-Rad). Elution of the His_6-tagged protein was carried out using a combined isocratic/gradient method with imidazole. Fractions were analyzed by SDS-PAGE (Criterion™ TGX stain-free precast gels, TGX buffer, Bio-Rad, 200 V, 45 minutes and imaged using a Bio-Rad ChemiDoc MP system). The combined fractions were mixed with Na_2EDTA (5 mM, 10 minutes) and concentrated to a volume of 1 mL (Amicon Ultra-15 centrifugal filter units, 50 kDa MW cut-off). The solution was diluted with buffer (20 mM TEOA, pH 7.5, 1:15) and concentrated to 1 mL, followed by a desalting step (PD-10 column, GE Healthcare) according to the supplier's manual. Aliquots were flash frozen in liquid nitrogen and stored at -20°C.

Size exclusion purification of *Sw*HKA WT and mutants: For crystallization purposes, the enzyme was further purified by size exclusion chromatography using a Superdex 200 Increase 10/300 GL column (GE Healthcare Bio-Sciences, 0.5 mL/min, 20 mM HEPES, pH 7.5).

Aldolase activity assay. Purified *apo-Sw*HKA (0.05 mg/mL) was incubated with NADH (0.5 mM), LDH (10 U/mL) in TEOA buffer (20 mM, pH 7.5) and the holoenzyme was formed by the addition of $MnCl_2$ (0.2 mM) and potassium phosphate (5 mM). The reaction was initiated by the addition of oxaloacetate (0.5 mM) to afford a final volume of 1 mL and the change in absorption was followed over time (λ = 340 nm, 25°C, 800 rpm, on a Cary 60 UV-Vis spectrometer (Agilent Technologies), equipped with a TC1 stirring unit (Quantum Northwest)). Specific activities were calculated from the linear slopes and kinetic parameters were calculated by fitting the data with IGOR.

Determination of metal dissociation constants using activity as reporter:

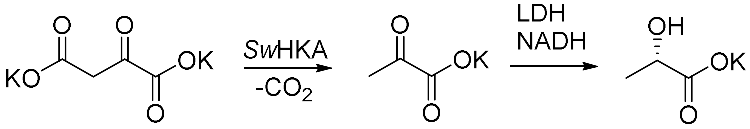

Scheme S1 | Coupled enzyme assay for the determination of retro-aldol activity. *Sw*HKA catalyzes the decarboxylation of oxaloacetate in a retro-aldol type reaction to afford pyruvate. Pyruvate is subsequently reduced by L-lactic dehydrogenase (LDH) to L-lactate and the concomitant consumption of NADH is followed spectrophotometrically at 340 nm. LDH is added in excess and the retro-aldol reaction is rate limiting in this assay.

Spectrophotometric assays were measured with a Cary 60 UV-Vis spectrometer (Agilent Technologies) equipped with a TC1 stirring unit (Quantum Northwest) in 1 mL PMMA cuvettes.

*Apo-Sw*HKA (0.05 mg/mL) was incubated with NADH (0.5 mM), LDH (10 U/mL) in TEOA buffer (20 mM, pH 7.5) and the holoenzyme was formed by the addition of the corresponding amounts of metal cofactor and inorganic phosphate (5 mM), respectively. The reaction was initiated by the addition of oxaloacetate (0.5 mM) to afford a final volume of 1 mL and the

change in absorption was followed over time (λ = 340 nm, 25°C, 800 rpm). Specific activities were calculated from the linear slopes and kinetic parameters were calculated by fitting the data to the equation for saturation kinetics with IGOR pro:

$$v_0 = \frac{[M^{2+}]*v_{sat}}{K_d+[M^{2+}]} \quad (1)$$

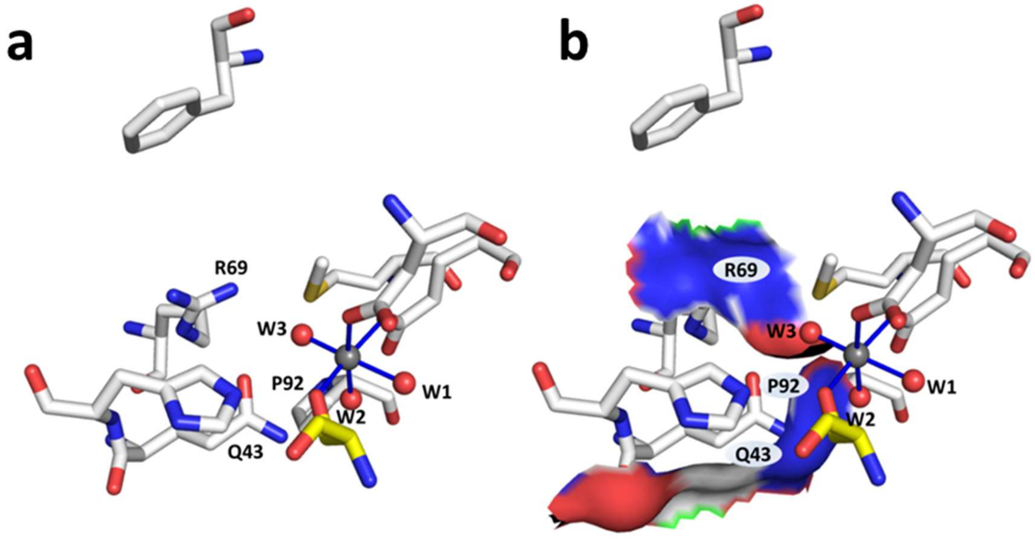

Fig. S1 | a, Structural clashes in M^{2+}_{R} with residues Q43, R69 and P92 do not allow for the exchange of W2 and W3 for the coordination of substrates at the bottom side of the octahedron. W1 is confined to the back end of the active site pocket by the presence of the metal cofactor and cannot be exchanged by a substrate. **b,** surface view of relevant residues. Figures were made with PyMol from PDBID 7NUJ (1.9 Å).

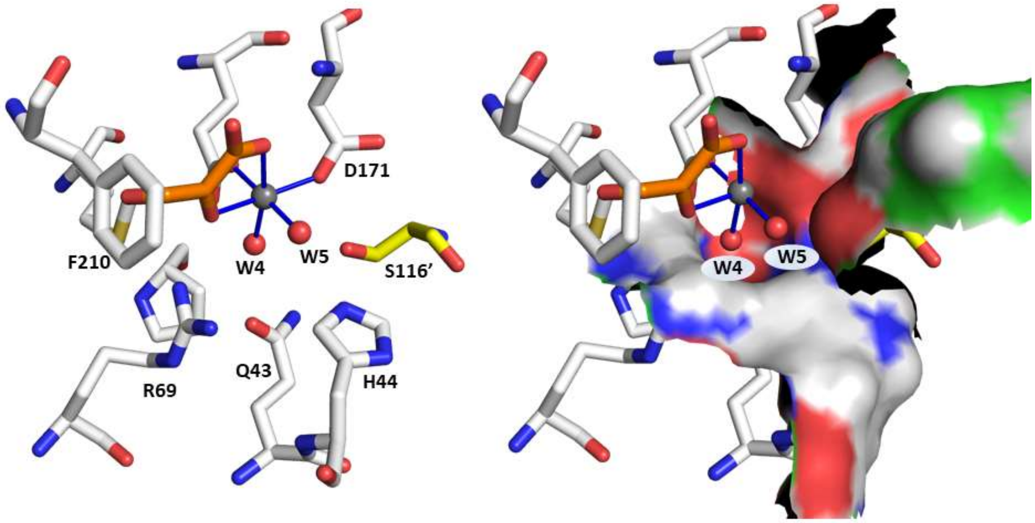

Fig. S2 | a, Structural clashes in $M^{2+}_{A,S}$ with H44, R69 and F117' prevent the exchange of water molecules W4 and W5 for the coordination of substrates in different configurations. **b,** Surface view of relevant residues. Figures were made with PyMol from PDBID 6R62 (1.55 Å).

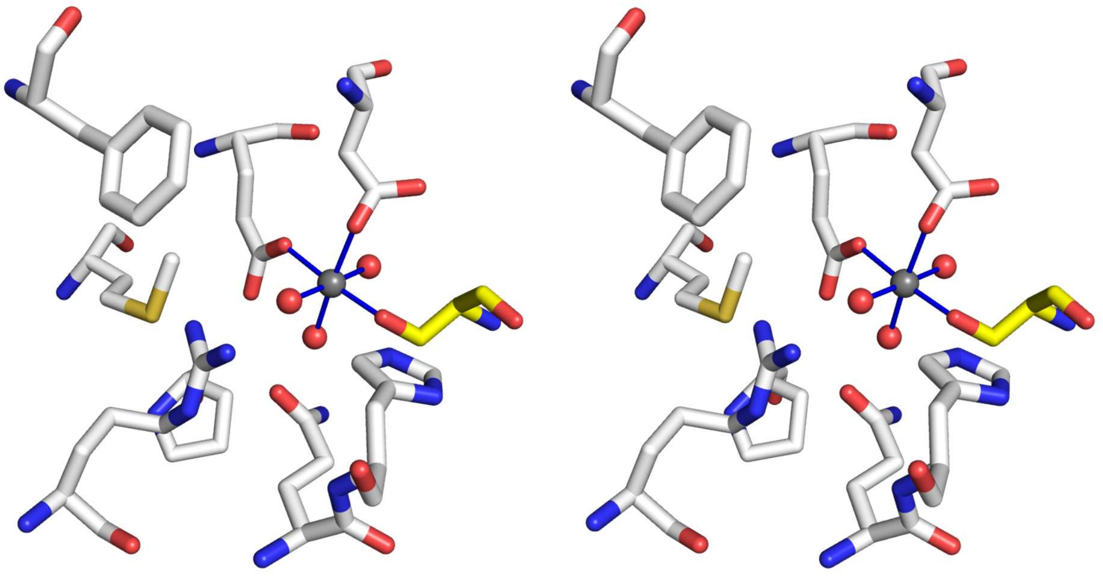

Fig. S3 | Wall-eye stereoview of Fig. 2a. The M^{2+}_{R} coordination sphere of the metal cofactor is located at the interface between two dimers (white, yellow, denoted by the prime symbol). Figures were made with PyMol from PDBID 7NUJ (1.9 Å).

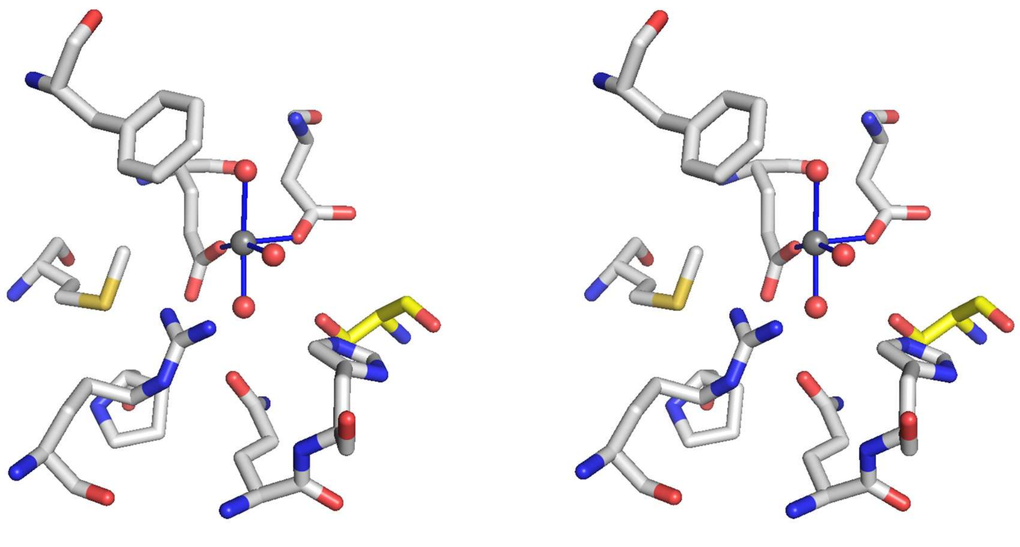

Fig. S4 | Wall-eye stereoview of Fig. 2b. Square pyramidal $M^{2+}_{A,W}$ coordination sphere with a vacant coordination site. This complex was observed with low occupancy in the absence of substrates (crystal structure PDBID 7O5R, 1.65 Å).

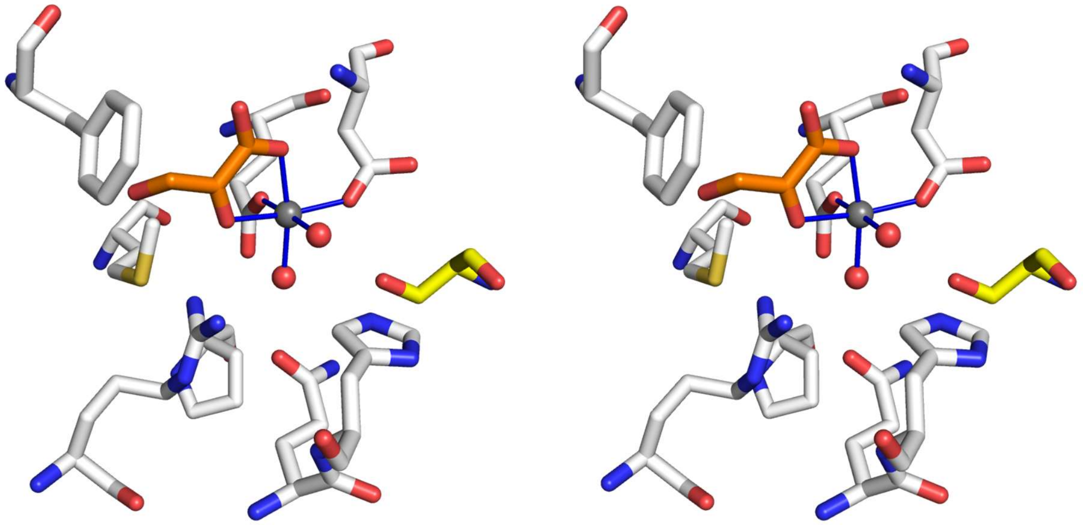

a)

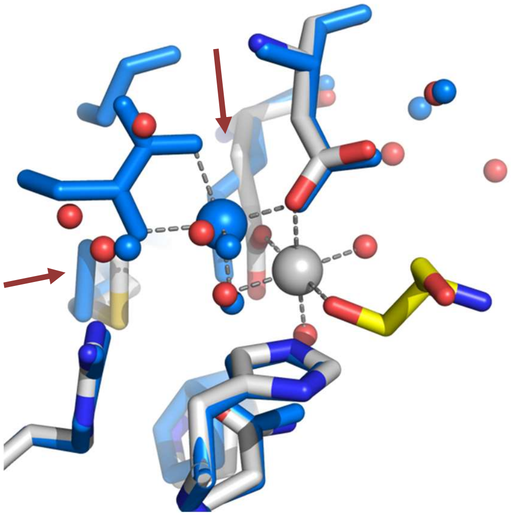

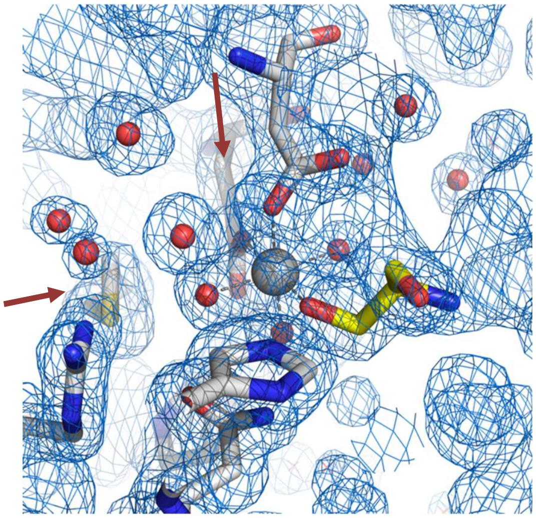

b) **c)**

Fig. S5 | a, Wall-eye stereoview of Fig. 2c. Coordination sphere of the catalytically active state $M^{2+}_{A,S}$. This complex was observed in the presence of hydroxypyruvate in crystal structures 6R62.pdb (1.55 Å), PDBID 7O87 (1.2 Å) and S116A-SwHKA-HPA PDBID 7NNK (1.8 Å). **b,** Structure superposition of *Sw*HKA-holo (7NUJ) M^{2+}_{R} configuration (Fig. **2a**) and *Sw*HKA-HPA (6R62) in M^{2+}_{AS} configuration (**Fig. 2c**) (Coloured in blue). **c,** Detailed view of the

metal center in *Sw*HKA-holo (7NUJ) (M^{2+}_{R} configuration) with the electron density map (2FoFc) contoured at 1.0 sigma level. Arrows (coloured in orange) show the two residues (Met 143 and E145) that experienced minor shifts (*ca.* 1.0Å).

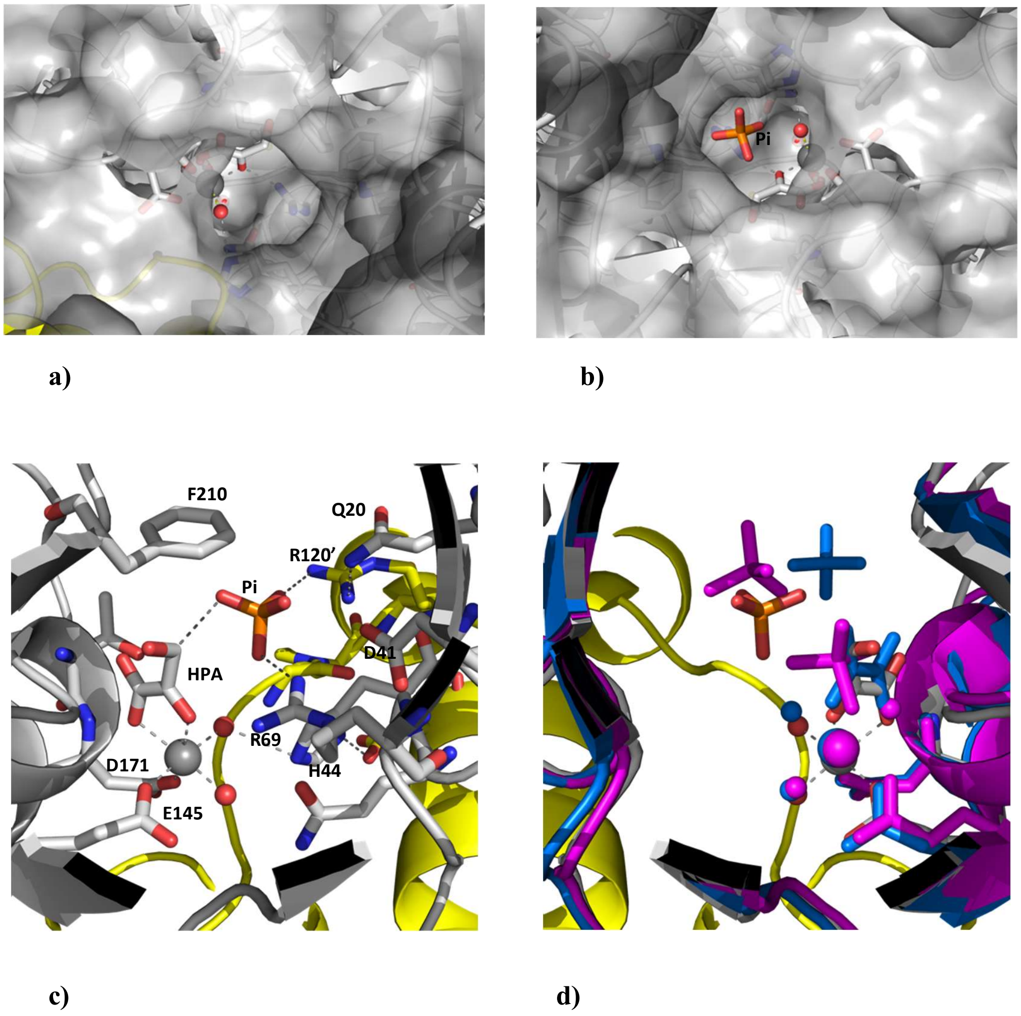

Fig. S6 | Phosphate docking site. a, Molecular surface around the active site highlighting an accessible channel. **b,** Representation of the highest score solution from the docking calculations with the $HPO4^{2-}$ onto *Sw*HKA-HPA (PDBID 6R62) molecular surface. **c,** Detail of the interaction made by the $HPO4^{2-}$ highest score solution in the active site of *Sw*HKA. **d,** Structural alignment between *Sw*HKA-HPA (PDBID 6R62) with the docked $HPO4^{2-}$ ion (coloured as in c) with *Escherichia coli* DDG aldolase (PDBID 1DXE, coloured in magenta) and *E. coli* Hpal aldolase (PDBID 4B5S, coloured in blue).

Table S1 | Overview of metal complexes and crystal structure resolutions.

$M^{2+}{}_{R}$		$M^{2+}{}_{A,S}$		$M^{2+}{}_{A,W}$	
7NUJ	1.90 Å	6R62	1.55 Å	7O5R	1.65 Å
7O9R	1.85 Å	7O5W	1.20 Å		
7O87	1.50 Å				

Table S2 | Overview of relative occupancies in *Sw*HKA crystal structures.

M^{2+}	$M^{2+}{}_{R}$		$M^{2+}{}_{A,W}$		$M^{2+}{}_{A,S}$[a]		pdb ID
	mol A	mol B	mol A	mol B	mol A	mol B	
Mg^{2+}	1	-[b]	0	-[b]	-[c]	-[c]	7NUJ
Mg^{2+}	0	-[b]	0	-[b]	1	-[b]	6R62
Mg^{2+}	1	0.3	0	0	0	0.7	7OBU
Mn^{2+}	0.61	0.76	0.36	0	n.a.	n.a.	7O5R

[a] Hydroxypyruvate was used as substrate for soaking studies. [b] The asymmetric unit is occupied by a monomer (mol A) in the space group $P4_232$. No dimer (A and B) was observed (different crystal form space group H3). [c] Crystals were soaked with the metal in the absence of substrate, excluding the formation of the $M^{2+}{}_{A,S}$ complex.

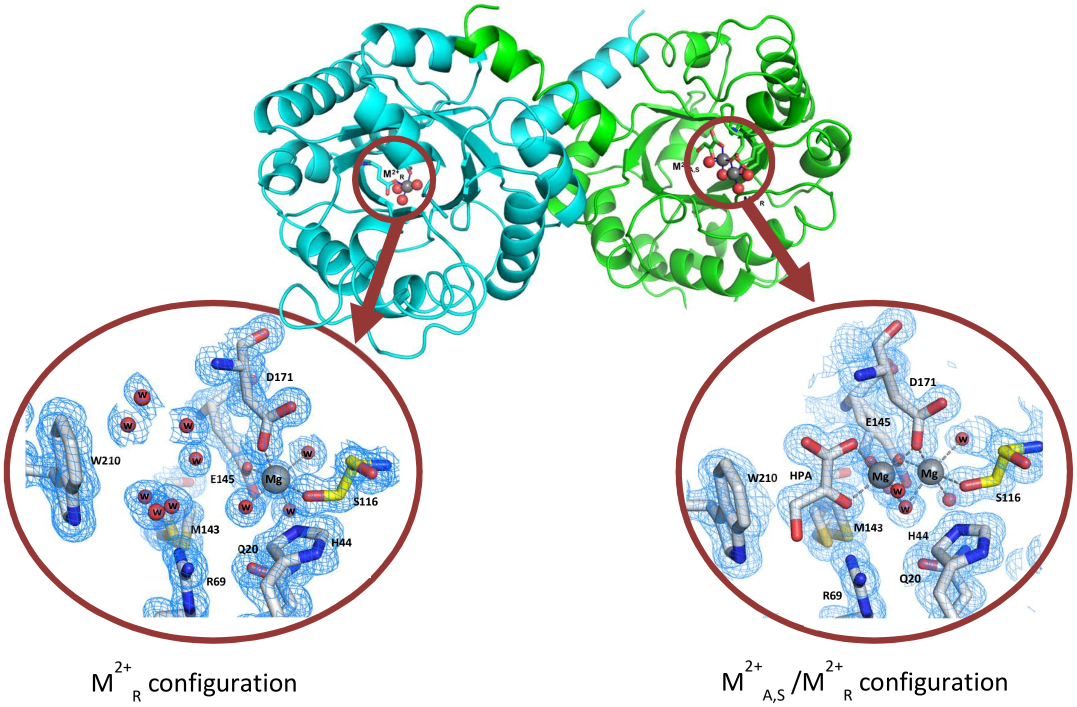

Fig. S7 | Crystal structure of the F210W variant of *Sw*HKA (PDBID 7OBU, 1.2 Å resolution). A dimer was observed in the asymmetric unit of F210W *Sw*HKA in the presence of $MgCl_2$ and hydroxypyruvate. Molecule A (cyan) exclusively shows the metal cofactor in the M^{2+}_R configuration (occupancy = 1.0), whereas molecule B (green) shows both $M^{2+}_{A,S}$ (occupancy = 0.7) and M^{2+}_R (occupancy = 0.3). Alignments and figures were made with PyMol. Enlarged view of the two different metal centers (M^{2+}_R configuration, $M^{2+}_{A,S}$ /M^{2+}_R configuration) with electron density map (2FoFc) contoured at 1.0 sigma level.

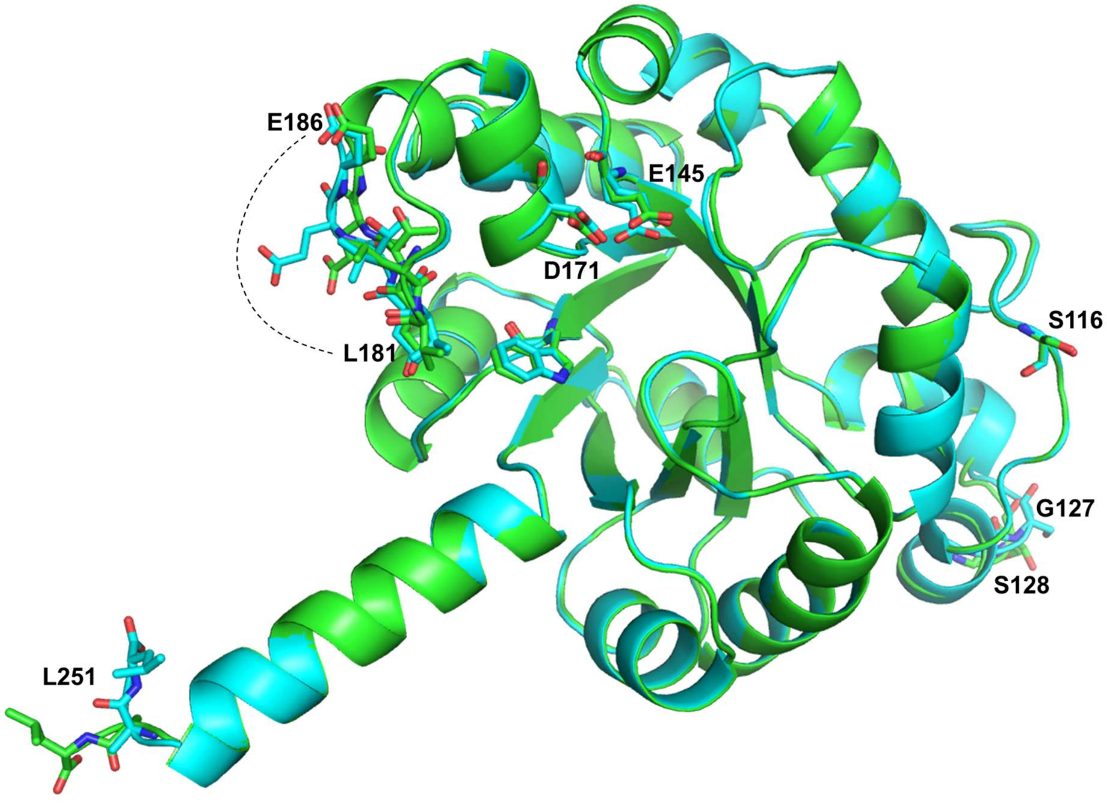

Fig. S8 | Alignment of molecules A (cyan) and B (green) in variant F210W (PDBID 7OBU, 1.2 Å resolution). Mobile, solvent exposed loops around residues L181-E186, G127, S128 and L251 constitute the main difference, while the overall structure of molecules A and B is largely identical (rmsd = 0.154 Å between 203 Cα atoms). A movement of residue E145 by 1.3 Å between M^{2+}_{R} and $M^{2+}_{A,S}$ was observed in this structure, whereas the change is more subtle for the other coordinating residues S116 and D171. These observations do not seem to support communication between the active sites in *Sw*HKA. Alignments and figures were made with PyMol.

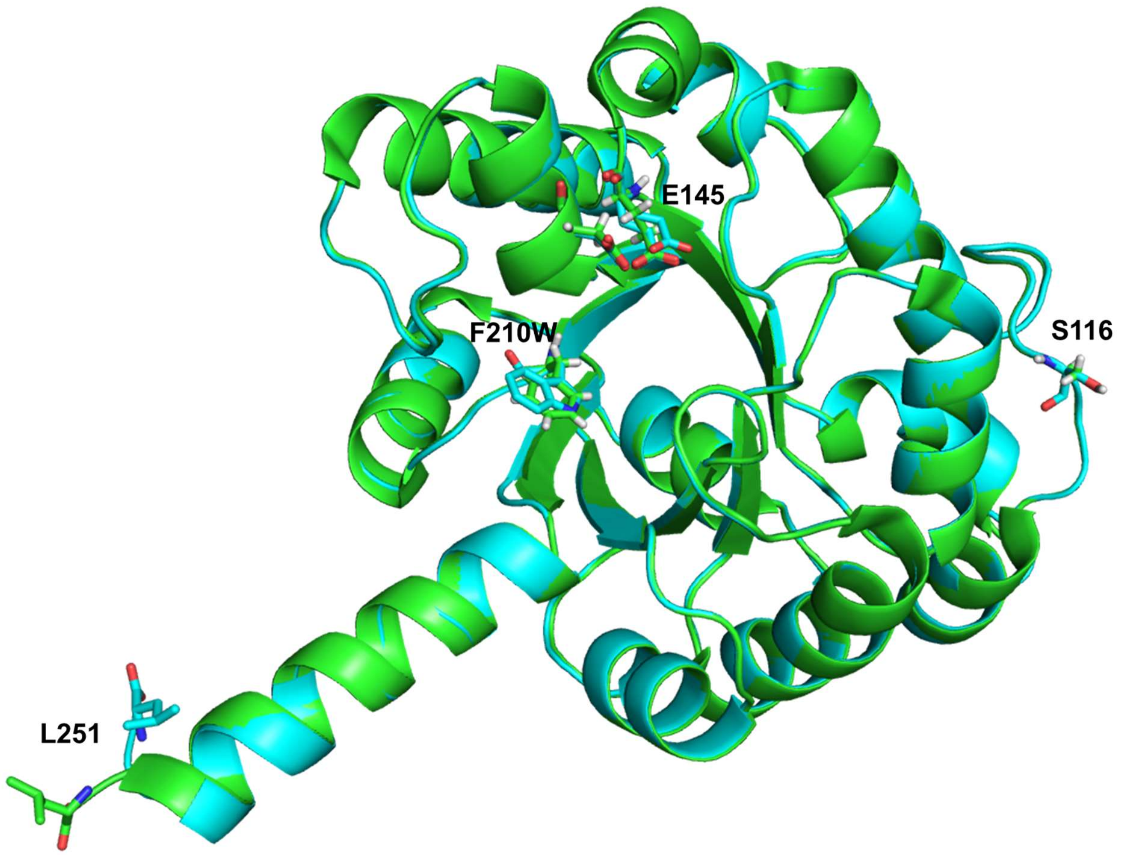

Fig. S9 | Alignment of WT *Sw*HKA $Mg^{2+}_{A,S}$ (green, PDBID 6R62) and molecule B in F210W *Sw*HKA containing $Mg^{2+}_{A,S}$ (cyan, PDBID 7OBU) (rmsd = 0.190 Å between 243 Cα atoms)). The positions of side chain residues in *Sw*HKA are unaffected by the mutation F210W. The lower occupancy of $Mg^{2+}_{A,S}$ in F210W (0.7) compared to WT *Sw*HKA (1.0) therefore does not seem to have structural reasons, but presumably originates from electronic interactions (such as CH-π) interactions with W210. F210W *Sw*HKA shows residue E145 in the position of M^{2+}_{R} with an occupancy of 0.3, next to a solvent exposed residue L251 as the only differences between both structures. Alignments and figures were made with PyMol.

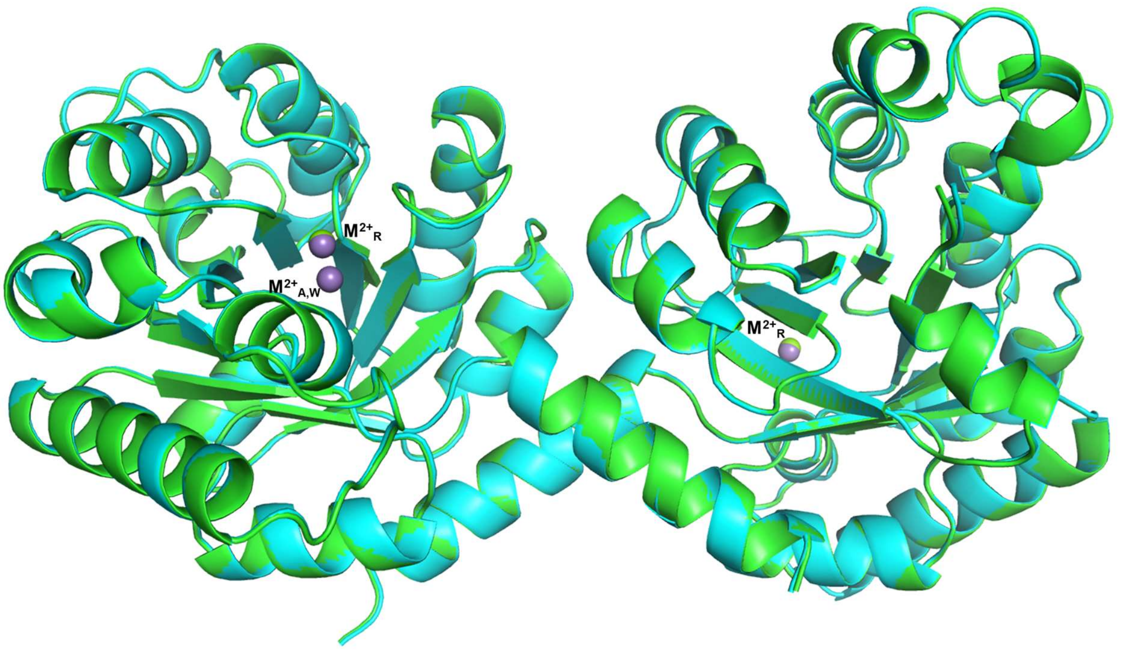

Fig. S10 | Alignment of WT *Sw*HKA Mg^{2+}_R (PDBID 7NUJ) and WT *Sw*HKA $Mn^{2+}_{A,W}/Mn^{2+}_R$ (PDBID 7O5R), rmsd = 0.195 Å between 210 Cα atoms. The use of either $MgCl_2$ or $MnCl_2$ for holoenzyme formation does not alter the overall protein structure. The mixed occupancy of $Mn^{2+}_{A,W}$ and Mn^{2+}_R in WT *Sw*HKA (and therefore the relative energy difference between the corresponding metal complexes) is therefore determined by the respective choice of metal, and not due to possible changes in protein structure. Alignments and figures were made with PyMol.

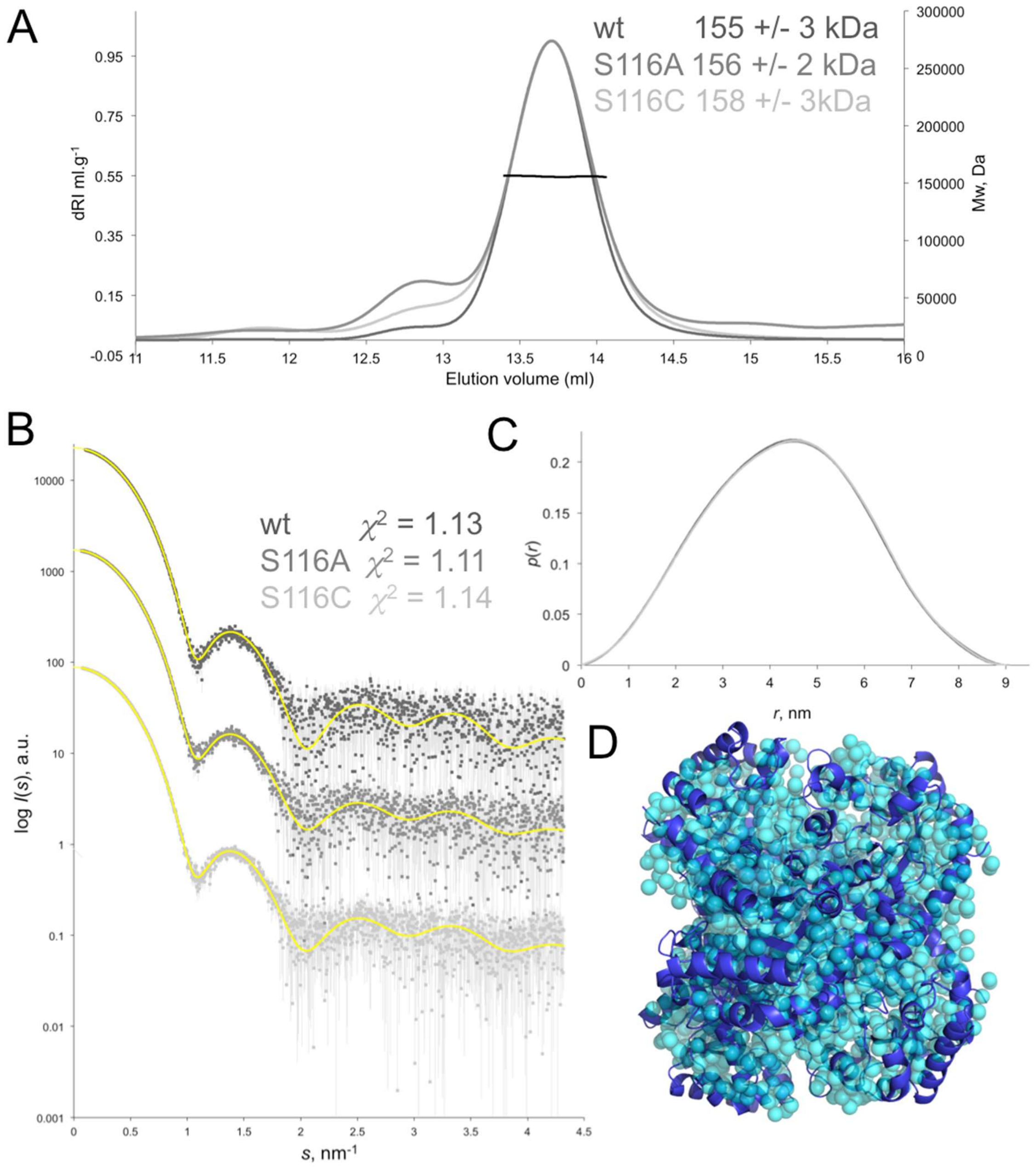

Fig. S11 - SEC-SAXS-MALLS results from wild-type *Sw*HKA (wt, dark grey), S116A (medium grey) and S116C (light grey) mutants. A. SAXS-coupled MALLS molecular weight correlations and averaged estimates for each aldolase variant determined through the predominant SEC elution peak of each sample (monitored using differential refractive index,

dRI). All three aldolase variants form hexamers (expected MW, hexamer: 165 kDa). B. The resulting SAXS profiles determined from the major elution peak of each aldolase variant and the corresponding fit to the data of the hexameric X-ray crystal structures (yellow). For clarity, the scattering intensities have been scaled on the I(s) axis. C. An overlay of the p(r) profiles calculated from the SAXS data showing a high-level of correspondence, and thus minimal perturbation, in the overall disposition of the aldolase hexamers caused by the S116A or S116C single-point mutations. D. A representative GASBOR ab initio model determined from the SAXS data (in this instance wt-*Sw*HKA, transparent spheres), spatially aligned to the high-resolution X-ray structure of the hexametric assembly as observed in the extended crystal lattice (blue ribbons; for fits to the data refer to B).

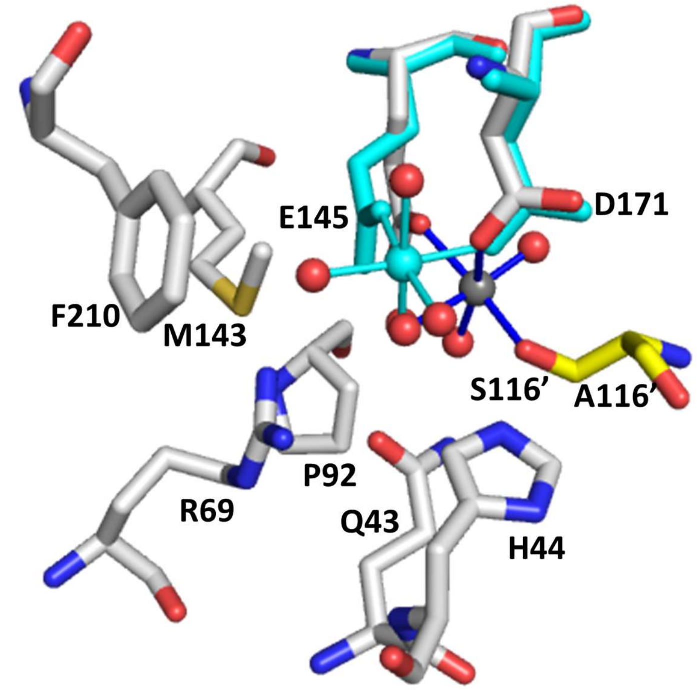

Fig. S12 | Alignment of WT *Sw*HKA Mg^{2+}_{R} (white/gray, PDBID 7NUJ**) and S116A *Sw*HKA $Mg^{2+}_{A,W}$ (cyan, PDBID** 7NR1**), rmsd = 0.115 Å between 230 Cα atoms.** Mutation of S116’ to A116’ prevents the formation of the catalytically inactive resting state Mg^{2+}_{R}, and affects the predominant occupation of $Mg^{2+}_{A,W}$ in the absence of substrates. A fourth water ligand completes an octahedral coordination geometry for $Mg^{2+}_{A,W}$ with an occupancy of 0.8. This suggests an equilibrium between square-pyramidal (three water ligands) and octahedral (four water ligands) configurations in S116A-*Sw*HKA-Mg (PDBID 7NR1).

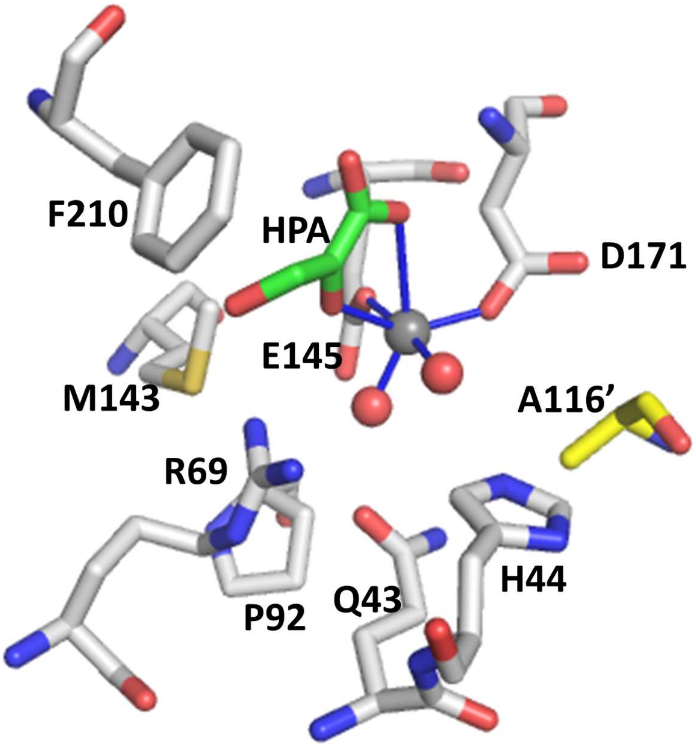

Fig. S13 | $M^{2+}_{A,S}$ complex in S116A *Sw*HKA HPA (PDBID 7NNK**).** The metal substrate complex showed a strangely distorted coordination geometry, which might explain its different catalytic behavior (Table 1).

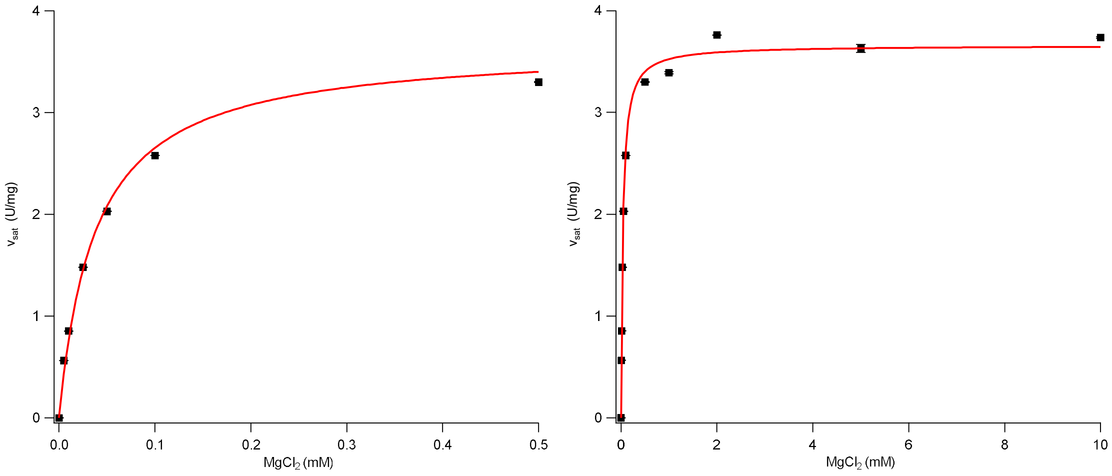

Fig. S14 | Metal saturation curve for mutant S116A with $MgCl_2$ in the absence of phosphate. Conditions: enzyme (0.05 mg/mL), $MgCl_2$ (0-10 mM), NADH (0.5 mM), LDH (10 U/mL), oxaloacetate (0.5 mM), in TEOA buffer (1 mL, 20 mM, pH 7.5, 25°C, 800 rpm). For clarity two figures with separate [$MgCl_2$] scales are shown.

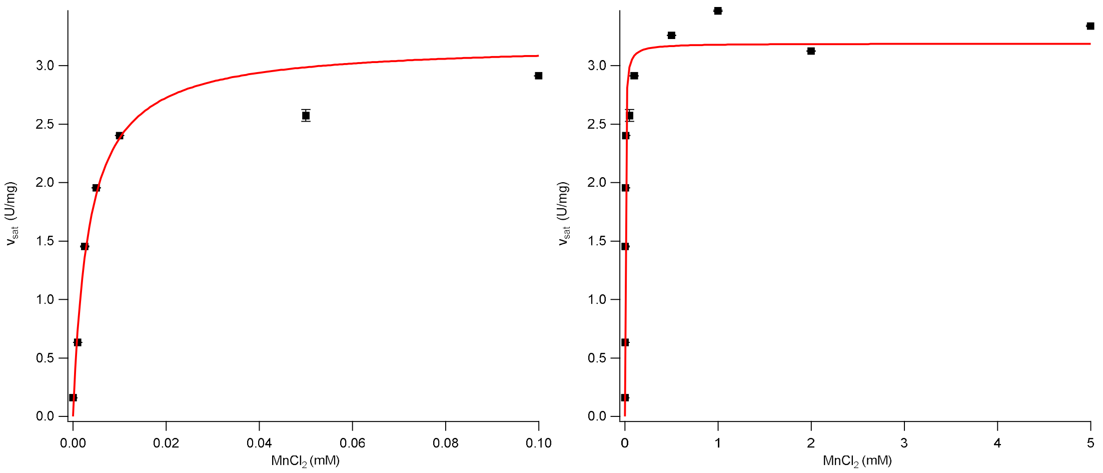

Fig. S15 | Metal saturation curve for mutant S116A with $MnCl_2$ in the absence of phosphate. Conditions: enzyme (0.05 mg/mL), $MnCl_2$ (0-5 mM), NADH (0.5 mM), LDH (10 U/mL), oxaloacetate (0.5 mM), in TEOA buffer (1 mL, 20 mM, pH 7.5, 25°C, 800 rpm). For clarity two figures with separate [$MnCl_2$] scales are shown.

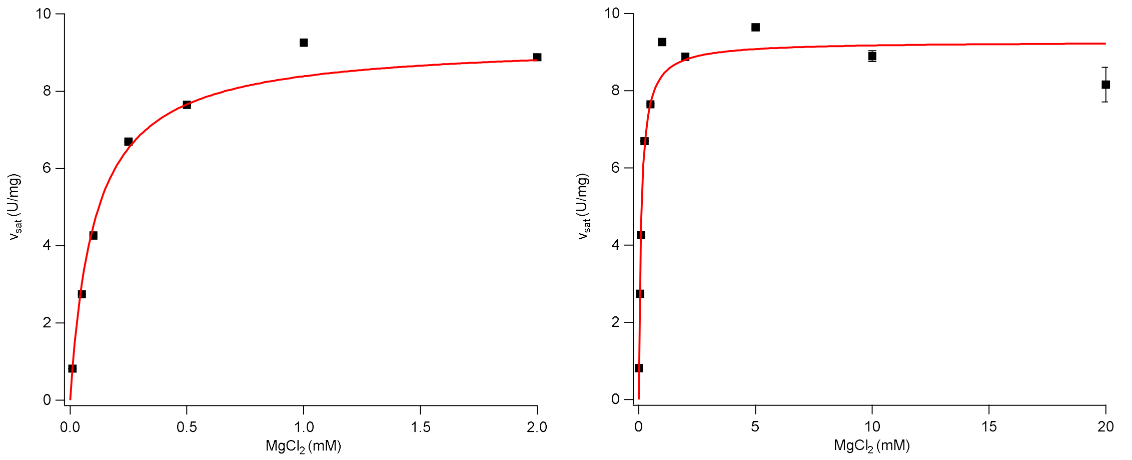

Fig. S16 | Metal saturation curve for mutant S116A with $MgCl_2$ in the presence of phosphate. Conditions: enzyme (0.05 mg/mL), $MgCl_2$ (0-20 mM), KPi (5 mM), NADH (0.5 mM), LDH (10 U/mL), oxaloacetate (0.5 mM), in TEOA buffer (1 mL, 20 mM, pH 7.5, 25°C, 800 rpm). For clarity two figures with separate [$MgCl_2$] scales are shown.

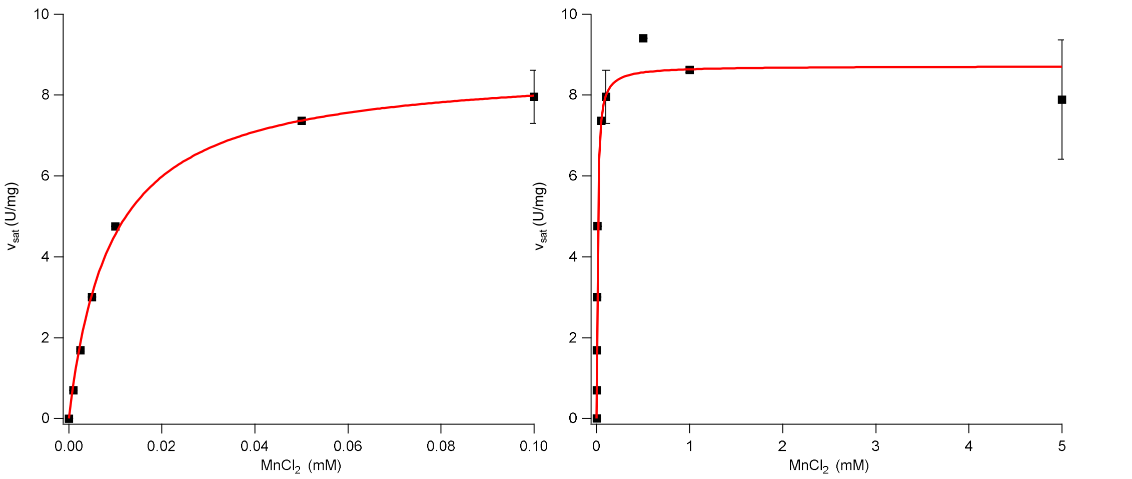

Fig. S17 | Metal saturation curve for mutant S116A with $MnCl_2$ in the presence of phosphate. enzyme (0.05 mg/mL), $MnCl_2$ (0-5 mM), KPi (5 mM), NADH (0.5 mM), LDH (10 U/mL), oxaloacetate (0.5 mM), in TEOA buffer (1 mL, 20 mM, pH 7.5, 25°C, 800 rpm). For clarity two figures with separate [$MnCl_2$] scales are shown.

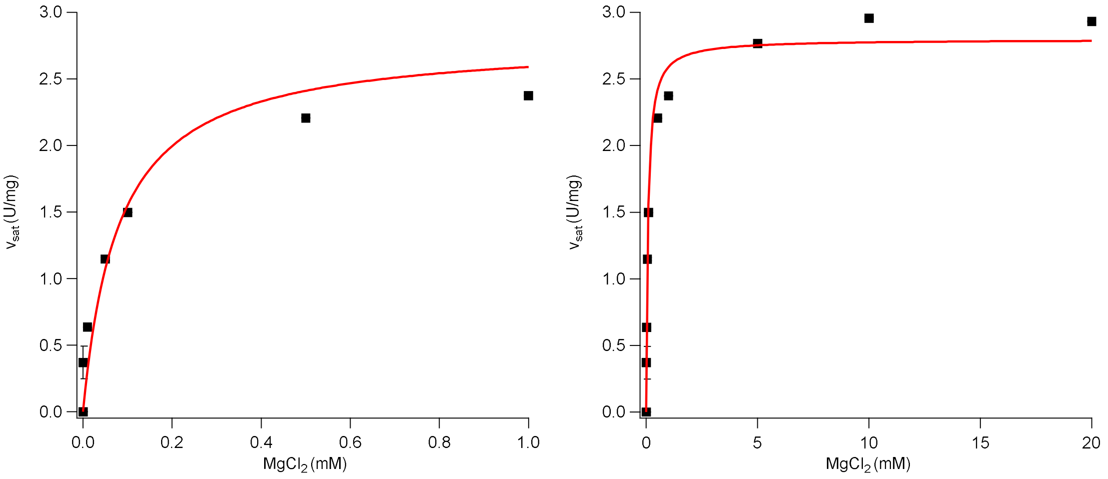

Fig. S18 | Metal saturation curve for mutant S116C with $MgCl_2$ in the absence of phosphate. Conditions: enzyme (0.05 mg/mL), $MgCl_2$ (0-20 mM), NADH (0.5 mM), LDH (10 U/mL), oxaloacetate (0.5 mM), in TEOA buffer (1 mL, 20 mM, pH 7.5, 25°C, 800 rpm). For clarity two figures with separate [$MgCl_2$] scales are shown.

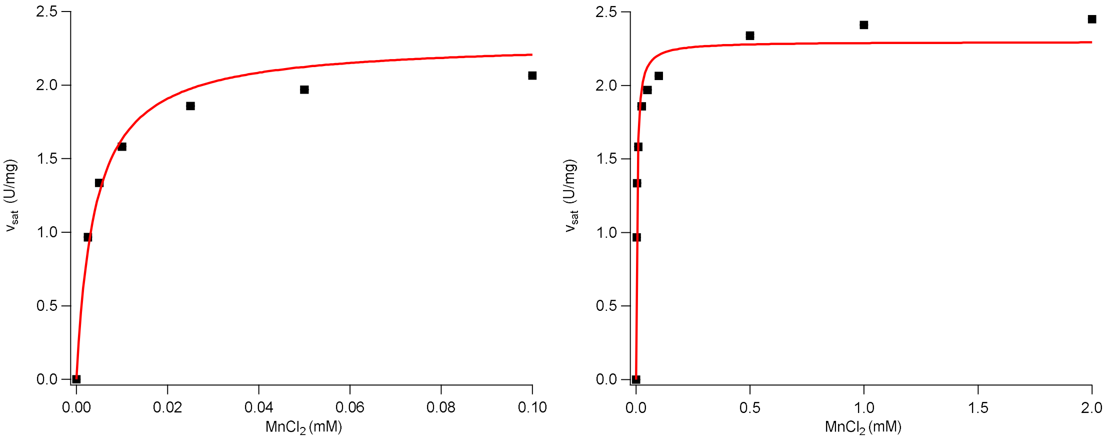

Fig. S19 | Metal saturation curve for mutant S116C with $MnCl_2$ in the absence of phosphate. Conditions: enzyme (0.05 mg/mL), $MnCl_2$ (0-2 mM), NADH (0.5 mM), LDH (10 U/mL), oxaloacetate (0.5 mM), in TEOA buffer (1 mL, 20 mM, pH 7.5, 25°C, 800 rpm). For clarity two figures with separate [$MnCl_2$] scales are shown.

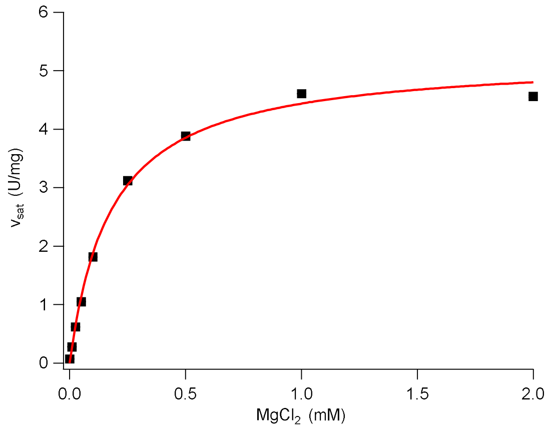

Fig. S20 | Metal saturation curve for mutant S116C with $MgCl_2$ in the presence of phosphate. Conditions: enzyme (0.05 mg/mL), $MgCl_2$ (0-20 mM), KPi (5 mM), NADH (0.5 mM), LDH (10 U/mL), oxaloacetate (0.5 mM), in TEOA buffer (1 mL, 20 mM, pH 7.5, 25°C, 800 rpm). For clarity two figures with separate [$MgCl_2$] scales are shown.

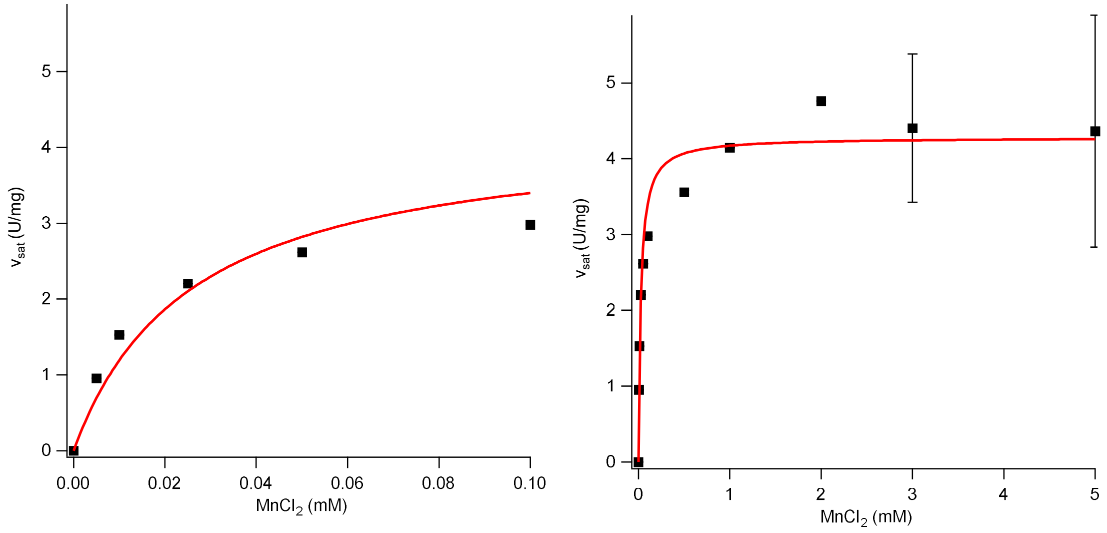

Fig. S21 | Metal saturation curve for mutant S116C with $MnCl_2$ in the presence of phosphate. Conditions: enzyme (0.05 mg/mL), $MnCl_2$ (0-5 mM), KPi (5 mM), NADH (0.5 mM), LDH (10 U/mL), oxaloacetate (0.5 mM), in TEOA buffer (1 mL, 20 mM, pH 7.5, 25°C, 800 rpm). For clarity two figures with separate [$MnCl_2$] scales are shown.

EPR measurements. EPR measurements were carried out using a Bruker EMXplus 9.5 spectrometer and the following conditions: 9.402 GHz microwave frequency, 0.2 mW microwave power, 100 kHz modulation frequency, 10 Gauss modulation amplitude at a temperature of 37 K. The microwave power was optimized by recording a 2D powerplot from 0 to 40 dB using the Xenon software (Bruker), which was analyzed using a labview EPR analysis program written by W.R. Hagen (to be published elsewhere). The low temperature was maintained by boiling liquid helium and the cold helium vapor was passed through a double wall quartz glass tube which was mounted and fitted in the rectangular cavity.[2,3] Samples were prepared in TEOA buffer (5 mM, pH=7.5) to contain 1.2 mM of purified *apo*-enzyme (calculated for the molecular weight of the monomer of 27.4 kDa), Mn^{2+} (1 mM, 0.85 eq.), KPi (2 mM, 1.7 eq.) ketoacids/aldehyde substrate (10 mM, 8.3 eq.) to afford a final volume of 200 μL.

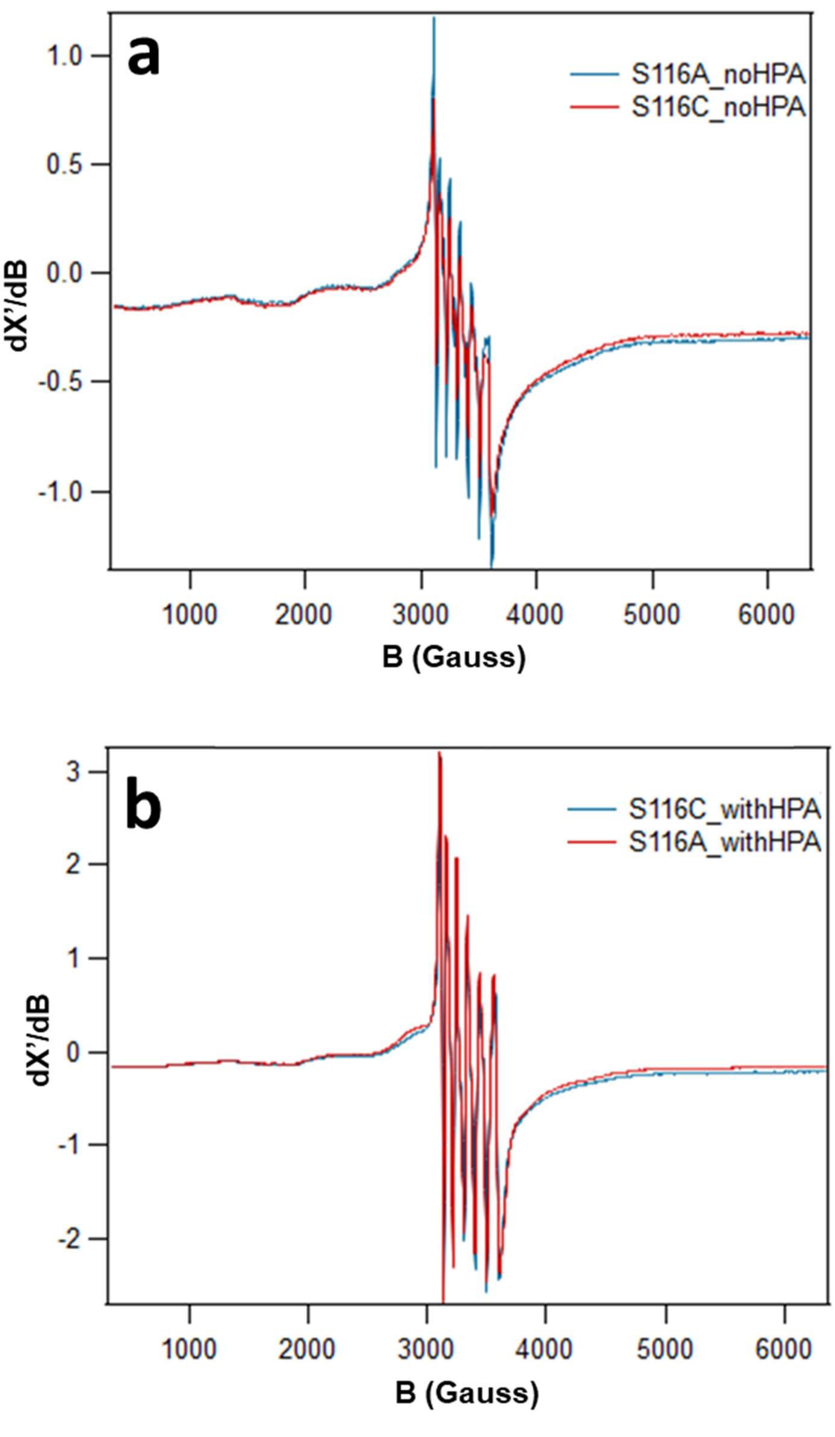

Fig. S22 | EPR spectrums of *Sw*HKA variants S116A and S116C. EPR conditions: EPR measurements were carried out using a Bruker EMXplus 9.5 spectrometer and the following conditions: 9.402 GHz microwave frequency, 0.2 mW microwave power, 100 kHz modulation frequency, 10 Gauss modulation amplitude at a temperature of 37 K.

The microwave power was optimized by recording a 2D powerplot from 0 to 40 dB using the Xenon software (Bruker), which was analyzed using a labview EPR analysis program written by W.R. Hagen (to be published elsewhere). The low temperature was maintained by boiling liquid helium and the cold helium vapor was passed through a double wall quartz glass tube which was mounted and fitted in the rectangular cavity.[2,3] Samples were prepared in TEOA buffer (5 mM, pH=7.5) to afford a final volume of 200 µL and contained **a,** 1.2 mM of purified *apo*-enzyme (calculated for the molecular weight of the monomer of 27.4 kDa), Mn^{2+} (1 mM, 0.85 eq.), KPi (2 mM, 1.7 eq.) and **b,** 1.2 mM of purified *apo*-enzyme (calculated for the molecular weight of the monomer of 27.4 kDa), Mn^{2+} (1 mM, 0.85 eq.), KPi (2 mM, 1.7 eq.) hydroxypyruvate (10 mM, 8.3 eq.).

Determination of dissociation constants by isothermal calorimetry measurements (ITC). The ITC cell (VP-ITC MicroCal, Malverne) was filled with a stock solution of apo-*Sw*HKA (2 mL, 8.1 mg/mL in 20 mM TEOA, pH 7.5, ≈ 300 µM corresponding to approximately 50x K_d) and titrated against a $MnCl_2$ solution (2.54 mM, 20 mM TEOA, pH 7.5) using identical buffers with the following parameters: number of injections: 28, cell temperature: 25°C, initial delay: 60 seconds. A control experiment was performed to correct for the integrated heat of dilution by titrating the $MnCl_2$ solution into the buffer.

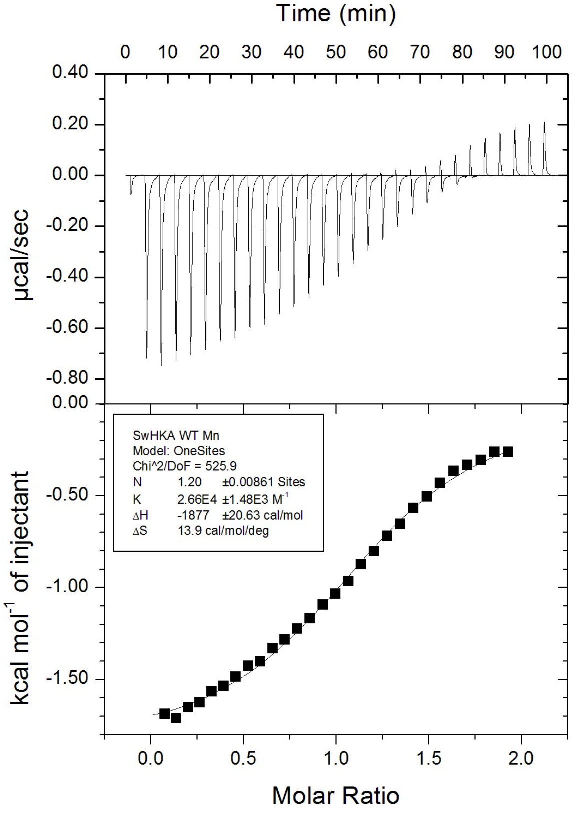

Fig. S23 | ITC curves for the binding of $MnCl_2$ to WT *apo-Sw*HKA. A dissociation constant of 37.6 µM was determined. Conditions: *apo-Sw*HKA (8.1 mg/mL) in 20 mM TEOA, pH 7.5 titrated against $MnCl_2$ (2.54 mM in identical buffer), 25°C, 28 injections, initial delay: 60 seconds.

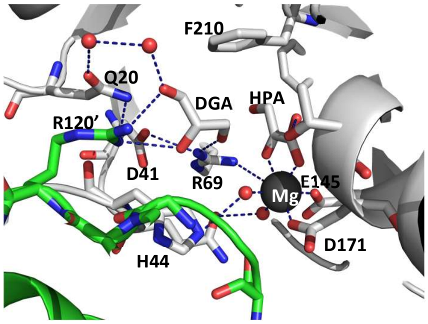

a)

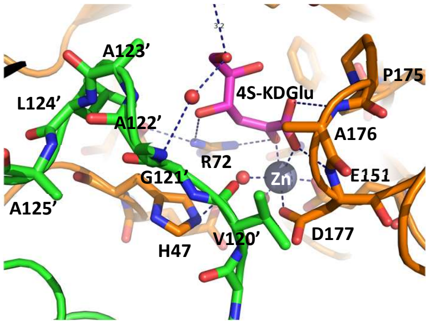

b)

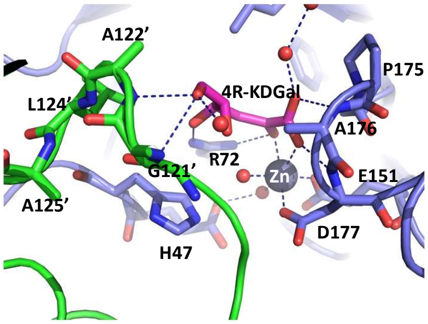

c)

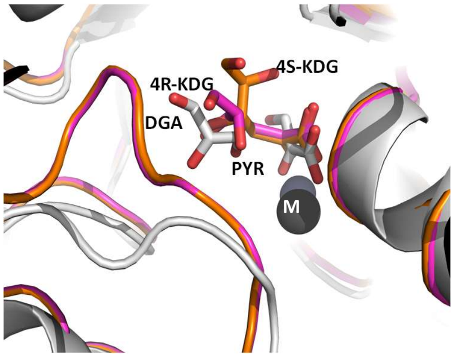

d)

Fig. S24 | Enlarged view of the active site with substrates or products bound. a, *Sw*HKA (PDBID 8ADQ) one monomer grey the other green. **b,** *Ab*HPA-(4*S*)-KDG (PDBID 7ETD) one monomer orange the other green. **c,** *Ab*HPA-(4*R*)-KDG (PDBID 7ETC) one monomer blue the other green. **d,** Structural alignment of all three crystal structures *Sw*HKA in gray, *Ab*HPA-(4*S*)-KDGlu in orange, and *Ab*HPA-(4*R*)-KDGal in magenta. Waters molecules are represented by red spheres.

X-ray Crystallography

Protein crystals of *Sw*HKA-apo, *Sw*HKA-holo (with either Mg^{2+} or Mn^{2+}) and variants enzymes (S116A, F210W, H44A) were obtained using the vapor diffusion method and the hanging drop technique following the procedure described by Marsden *et al.* 2019.[1] In particular, *Sw*HKA-apo, *Sw*HKA-holo, F201W and H44A appeared at 277K from drops containing equal volumes of a protein sample at 7 mg/ml with a crystallization solution comprising of 0.44 M to 0.65 M of sodium citrate and 0.1 M HEPES (4-(2-hydroxyethyl)-1-piperazine ethanesulfonic acid) at pH 7.0. Crystals took *ca.* 7 days to appear and grew to their final size within a few days. All crystals were left on the crystallization drops for about three weeks prior being soaked with a solution containing 0.7 M of potassium bromide (KBr), 0.1 M HEPES pH 7.0 and 20mM of the desired ligands (HPA, Mg or Mn) and the cryo protecting agent (PEG 400 25%). The crystallization conditions were then exchanged by slowly replacing the mother liquid over a 30 minutes period with the new soaking solution containing the selected ligands at a concentration of 50 mM. The crystals were kept in these condition for another 30 minutes before flash frozen in liquid nitrogen. Crystals of the holo-S116A, on the other hand, were obtained at 292 K from drops resulting from mixing 1,8µl of a protein sample at 7 mg/ml (in 20mM HEPES, pH 7.5) with 1,2µl of a crystallization solution consisting of 20-30% of PEG400, 0.1-0.2 M $MgCl_2$ and 0.1 M HEPES at pH 7.50-8.00. Crystals appeared within 5 days, as described above, and were left in the crystallization drop for *ca.* three weeks before the soaking protocol was executed. In this case the soaking solution contained 30%PEG400, 0.1 M $MgCl_2$, 0.1 M HEPES at pH 7.50 and 50 mM of the target ligand (Mg^{2+} and (HPA)). Crystals were left in these conditions for another 15 minutes and then were flash frozen in liquid nitrogen. X-ray diffraction data sets were collected at beamlines P13 and P14 operated by EMBL Hamburg at the PETRA III storage ring (DESY, Hamburg, Germany).[4] The protein crystallized in two different space groups: cubic Apo-

SwHKA’ crystals (space group P 42 3 2), with one molecule in the asymmetric unit and a solvent content of 47.5%, similar to what was observed for the SwHKA-HPA (PDBID 6R62);[1] whereas the remaining enzymes (Holo-*Sw*HKA, H44A, S116A, F210W) crystallized in the hexagonal space group with two molecules per asymmetric unit. Data were indexed and integrated with XDS,[5] scaled with AIMLESS,[6] and the space group was determined with POINTLESS[7] from CCP4 programs suite.[8] Data-collection statistics are given in Table S4. The crystal structure of *apo*-*Sw*HKA was solved by S-SAD using SHELX programs[9] and HKL2MAP[10] and was then used as a search model for molecular replacement with the program MOLREP[11] for determination of the remaining crystal structures. Model building and refinement of all protein structures was performed using the programs COOT[12] and REFMAC[13], or PHENIX[14], respectively. All crystal structures were checked periodically using PDB_redo server (https://pdb-redo.eu/)[15] and validated with MOLPROBITY.[16] Structure refinement statistics are listed in Table S4.

Phosphate Docking

Both monohydrogen phosphate and dihydrogen phosphate ions were docked in PDBID 6R62 with AutoDock Vina 1.2.0.[17] The structure was protonated with AutoDockTools using standard protonation states (Asp and Glu as negatively charged residues, Arg and Lys as positively charged residues and His protonated in the epsilon nitrogen with neutral charge) while the ligand was prepared with PyMol building tool. Water molecules were removed from the structure, except those coordinating the Mg cation. The exhaustiveness of 8 and a box size of 30x30x30.

Small Angle X-ray Scattering

Small angle X-ray scattering (SAXS) coupled to size exclusion chromatography and multi-angle laser light scattering (SEC-SAXS-MALS) was performed on WT-*Sw*HKA and the S116A and S116C aldolase mutants at the EMBL bioSAXS-P12 beam line at PETRAIII, Hamburg, Germany.[18] The SAXS intensities, I(s) vs s, (where $s = 4\pi \sin\theta/\lambda$, 2θ is the scattering angle and λ= 0.124 nm) were measured from the continuously flowing SEC column eluate (25°C; 0.6 ml/min; GE Healthcare S200 Increase 10/300), where the flow-stream containing the separated components of each sample was split evenly between the SAXS beam line and additional UV (280 nm), three-angle MALS, quasi-elastic light scattering (QELS) and refractive index (RI) detectors (λ, 659 nm; Wyatt technologies.[19] The SEC running buffer was 20 mM HEPES, 100 mM NaCl, 20 mg/l $MgCl_2$, pH 7.5, where the injection concentration and volume were approximately 10 mg/ml and 75 μl, respectively. The SAXS data were recorded using a Pilatus 6M area detector as a set of successive 1s X-ray exposures spanning the entire column volume for each run (24 ml; 2800 data frames total). Automated data reduction steps were performed using the SASFLOW pipeline (2D-to-1D azimuthal averaging)[20] and the resulting SAXS chromatograms of the 1D integrated SAXS intensity vs. frame number were processed using CHROMIXS[21] that included the subtraction of the SEC-buffer scattering intensities from the sample frames and the estimation of the radius of gyration, Rg, through the SEC-peak using the Guinier approximation[22] (lnI(s) vs s2, for sRg < 1.3)). Those background-corrected sample data frames with a consistent Rg were scaled and averaged to generate the final SAXS profiles of each aldolase variant. Additional data evaluation and modelling was performed using the modules of the ATSAS package,[23] that included: SHANUM[24] to evaluate the useable smax of the data, taking into account the variance in I(s) and level of oversampling; GNOM[25] for the calculation of the probable distribution of real-space distances (p(r) profiles) and; DATBAYES[26] for the

estimation of the concentration-independent molecular weight (MW) and MW credibility interval determined from the scattering profiles. Both AMBIMETER[27] and DATCLASS[28] were used to assess the overall ambiguity and shape classification of the SAXS data, that then underwent subsequent ab initio dummy-residue modelling using GASBOR (in P32 symmetry).[29] CRYSOL was used to calculate the fit to the SAXS data of the high-resolution X-ray crystal structure of the aldolase hexamer.[30] The fit evaluations of both the GASBOR and crystal structure models were assessed using the reduced χ^2 test in combination with the Correlation Map (CorMap) P-value.[31] The SAXS data, p(r) profiles, models and respective model fits have been deposited to the Small Angle Scattering Biological Data Bank (SASBDB)[32] with the accession codes: SASDKS7 (WT-aldolase); SASDKT7 (S116A aldolase) and; SASDKU7 (S116C aldolase). The molecular weight estimates derived from the parallel MALLS/RI measurements, as well as the hydrodynamic radius, RH, modelled from the resulting QELS autocorrelation functions, were determined using Wyatt ASTRA7 software (RI dn/dc, 0.185 ml.g-1; solution viscosity, 0.890 cP). The UV-MALLS-QELS-RI data are made available in the respective SASBDB entries.

Table S3: SAXS data reporting Table for HpcH/HpaI aldolase

Sample details			
Organism		*Sphingomonas wittichii*	
Uniprot ID		A5VH82 (amino acids 2-251, plus N-term 6-His tag)	
Solvent composition		100 mM NaCl, 20 mg/L $MgCl_2$, pH 7.5	
Temperature (°C/K)		25/298	
Instrument details			
Instrument		EMBL-P12 BioSAXS	
X-ray wavelength, nm		0.124	
Sample to detector distance, m		3	
Sample path length, mm		1	
s-measurement range, nm^{-1}		0.03-7.27	
SEC-SAXS parameters:			
Column type		GE S200 Increase 10/300	
Flow rate, mL/min		0.6	
Injection volume, μl		75	
Load concentration, mg/mL		~10	
Exposure time/data frame, s		1	
Sample	**WT-SwHKA**	**S116A**	**S116C**
Information content:			
Method		SHANUM	
#data frames used for averaging	35	30	28
Final working *s*-range, nm^{-1}	0.10-4.3	0.068-4.3	0.07-4.3
Final #Shannon channels	12	13	13
Guinier analysis:			
Primary data analysis software		PRIMUSQT/*AUTORG*	
Guinier *I*(0) (σ), a.u	22638 (24)	17151(12)	17503 (12)
Guinier R_g, nm (σ)	3.31(0.07)	3.33(0.1)	3.32(0.1)
sR_g range	0.42-1.27	0.23-1.28	0.24-1.29
p(r) analysis:			
Method		GNOM5	
I(0), (σ), a.u.	22640(12)	17130(7)	17510 (8)
R_g, nm (σ)	3.27(0.01)	3.29(0.01)	3.28 (0.01)
D_{max}, nm	9	9.1	9
Quality of fit, CorMap *P* value/χ^2	0.76/1.07	0.51/1.05	0.30/1.04
Porod volume, nm^3	224	222	223
Shape classification	compact	compact	compact
MW and hydrodynamics:			
MW, from amino acid sequence		165 kDa (hexamer)	
**MW from MALLS/RI**	155 +/- 3	156 +/- 2	158 +/- 3
**MW from SAXS**			
Bayesian estimate, kDa	147	147	147
MW interval, kDa	142-163	142-151	134-163
**Hydrodynamics, QELS**			
Average R_h, nm (σ)	4.3(0.1)	4.4(0.3)	4.4(0.2)
R_g/R_h ratio	~0.77	~0.75	~0.75
Data availability/Accession code(s)			
SASBDB	SASDKS7	SASDKT7	SASDKU7

Nano-Differential scanning fluorimetry (nanoDSF). Nano-differential scanning fluorimetry (nDSF) technique was used to access the stability of the target enzymes. All experiments were done with Prometheus NT.48 (Nanotemper Technologies), between 15 and 95ºC, with a temperature increase rate of 1ºC/minute and at a fluorescence intensity of 50%. Assays were performed in triplicate and samples containing WT-*Sw*HKA, S116A and S116C variants at a concentration of 5 mg/mL, were incubated with a solution containing $MgCl_2$ (10 mM), hydroxypyruvate (20mM), and Hepes buffer (20 mM, pH 7.5) for 30 minutes prior measurement.

Table S4. Data collection and Refinement Statistics

	Apo-SwHKA	Holo-SwHKA-Mg	Holo-SwHKA-Mn	Holo-SwHKA-HPA-DGA	Holo-H44A	Holo-H44A-HPA	Holo-S116A	Holo-S116A-HPA	Holo-F210W	Holo-F210W-HPA	Holo-F210W-HPA
**PDB ID**	7O5I	7NUJ	7O5R	8ADQ	7O9R	7O5V	7NR1	7NNK	7O5W	7O87	7OBU
Data collection											
**X-ray diffraction source**	P13	P14	P13	P13	P13	P14	P13	P13	P13	P13	P13
**Wavelength (Å)**	0.9763	0.9766	0.9763	0.9763	0.9763	0.9766	0.9763	0.9763	0.9763	0.9763	0.9763
**Resolution range (Å)**	116.4 - 1.35 (1.37-1.35)*	59.36 - 1.90 (1.94-1.90)*	75.13 - 1.65 (1.68-1.65)*	49.94 – 1.60 (1.66-1.60)*	59.19 - 1.85 (1.89-1.85)*	53.77 - 1.95 (2.00-1.95)*	59.28 - 2.30 (2.38-2.30)*	74.39 - 1.80 (1.84-1.80)*	74.27 - 1.20 (1.22-1.20)*	73.97 - 1.50 (1.53- 1.50)*	35.55 - 1.20 (1.24- 1.20)*
**Space group**	P 42 3 2	H 3	H 3	P 42 3 2	H 3	H 3	H 3	H 3	H 3	H 3	H 3
**Unit cell (Å)/ (º)**	116.4	71.1 223.2	71.5 225.4	115.8	70.9 223.2	70.91 222.6	71.05 222.7	71.3 223.2	71.00 222.8	70.81 221.9	71.09 222.39
**Total Reflections**	1204977 (40504)*	175033 (11202)*	302866 (15530)*	1368942 (139193)*	127223 (8086)*	75644 (5426)*	159124 (10930)*	407073 (23014)*	1276169 (61070)*	213153 (10918)*	1223232 (113654)*
**Unique reflections**	59482 (2892)*	33130 (2122)*	51766 (2600)*	35573 (3490)*	33572 (2159)*	29321 (2104)*	18614 (1792)*	38705 (2312)*	130983 (6438)*	65270 (3274)*	131063 (13104)*
**Multiplicity**	20.3 (14.0)*	5.3 (5.3)*	5.9 (6.0)*	38.5 (39.9)*	3.8 (3.7)*	2.6 (2.6)*	8.5 (6.1)*	10.4 (10.0)*	9.7 (9.5)*	3.3 (3.3)*	9.3 (8.7)*
**Completeness (%)**	99.7 (100.0)*	99.9 (99.9)*	99.9 (100.0)*	99.96 (99.9)*	94.0 (97.7)*	96.3 (98.0)*	100.0 (99.9)*	100.0 (99.9)*	100.0 (100.0)*	98.2 (99.1)*	99.9 (99.8)*
**<I/σ(I)>**	12.9 (1.7)*	9.5 (1.4)*	9.1 (1.9)*	30.9 (1.3)*	4.6 (1.4)*	7.3 (1.0)*	7.5 (1.1)*	14.4 (1.8)*	15.5 (4.3)*	6.0 (1.5)*	10.5 (1.6)*
**R-merge**	0.11 (2.3)*	0.12 (1.3)*	0.16 (1.2)*	0.07 (2.9)*	0.13 (1.3)*	0.08 (0.8)*	0.20 (1.6)*	0.12 (1.4)*	0.08 (0.5)*	0.13 (0.9)*	0.12 (1.5)*
**$CC_{1/2}$**	0.99 (0.72)*	0.99 (0.58)*	0.99 (0.32)*	1.0 (0.65)*	0.99 (0.60)*	0.99 (0.38)*	0.99 (0.40)*	0.99 (0.60)*	0.99 (0.91)*	0.99 (0.38)*	0.99 (0.57)*

* Values at the highest resolution shell

	Apo-SwHKA	Holo-SwHKA-Mg	Holo-SwHKA-Mn	Holo-SwHKA-HPA-3GR	Holo-H44A	Holo-H44A-HPA	Holo-S116A	Holo-S116A-HPA	Holo-F210W	Holo-F210W-HPA	Holo-F210W-HPA
**PDB ID**	7O5I	7NUJ	7O5R	8ADQ	7O9R	7O5V	7NR1	7NNK	7O5W	7O87	7OBU
Refinement											
**Reflections used in refinement**	59220	33130	51781	35567	33316	29313	18592	38705	130974	65262	131014
**Reflections used for R-free**	2870	1657	2504	1766	1711	1484	910	1936	6604	3272	6618
**R factor**	0.138	0.164	0.162	0.166	0.158	0.162	0.215	0.183	0.118	0.184	0.129
**R free**	0.160	0.203	0.201	0.199	0.196	0.212	0.251	0.225	0.146	0.225	0.157
Number of non-hydrogen atoms											
**macromolecules**	1912	3819	3820	1906	3785	3747	3825	3800	3792	3827	3905
**ligands**	6	2	7	87	12	7	4	3	12	12	6
**solvent**	341	407	478	243	216	206	567	298	605	326	634
**Protein residues**	253	502	503	252	502	501	503	500	502	502	503
**RMS(bonds) (Å)**	0.016	0.014	0.013	0.011	0.014	0.016	0.071	0.016	0.017	0.015	0.008
**RMS(angles) (º)**	1.90	1.83	1.93	1.15	1.74	1.95	2.04	1.97	1.85	1.78	1.11
**Ramachandran favored (%)**	97.6	96.9	96.6	96.8	96.2	95.6	96.6	97.8	97.0	96.8	96.7
**Ramachandran outliers (%)**	0.40	0.40	0.20	0.40	0.20	0.0	0.60	0.00	0.40	0.40	0.40
**Rotamer outliers (%)**	0.00	0.52	0.51	0.00	0.26	1.33	0.52	1.31	0.36	0.78	0.25
**Clashscore**	3.32	2.09	5.74	1.80	2.76	4.39	3.00	0.92	3.14	2.47	1.78
**<B> (Å^2)**	24.1	28.2	14.3	35.3	29.0	35.1	18.6	24.1	13.3	18.5	13.4
**macromolecule**	21.6	27.4	13.2	34.2	29.7	34.9	17.2	23.6	11.5	17.8	11.8
**ligands**	38.6	30.2	14.1	49.4	42.2	34.7	19.5	24.1	19.5	23.8	13.6
**solvent**	36.7	35.1	22.9	40.9	32.9	37.3	28.1	30.5	24.3	25.8	23.1
**Metal**	n/a	Mg	Mn	Mg	Mg	Mg	Mg	Mg	Mg	Mg	Mg

QM energy calculations. Input structures for the theoretical calculations were generated from the corresponding X-ray structures. An octahedral active form with four water molecules was not observed during crystallography. Therefore, this hypothetical form was created from a substrate bound structure by converting the two coordinating substrate oxygens to water molecules. To evaluate whether hydroxyl ions or water molecules were present as ligands, the H-bonding network inside the active site was predicted with YASARA using the commands "CleanAll" and "OptHydAll".[33,34] There were no indications for hydroxyl ions, and therefore all coordinating water molecules were modeled as neutral. Input structures for the Mn^{2+} complexes were generated from the corresponding Mg^{2+} forms by replacing the metal ion. Calculations were performed with GAUSSIAN09.[35] The APFD and B3LYP-D3[36,37] methods were used separately to compare the results. In all cases, a 6-311++G(2d,p) basis set was used during both energy minimizations and frequency calculations. The solvent was modeled with a polarized continuum model. Because of the dense H-bonding network around the metal ligands inside the active site, water was selected as the solvent. An example of the applied keywords is "#p opt=(CalcFC,tight) freq b3lyp/6-311++g(2d,p) EmpiricalDispersion=GD3 scrf=(iefpcm,solvent=water) Int=UltraFine" (this was used for a Mg^{2+} complex modeled with restricted B3LYP-D3). For the Mn^{2+} complexes, separate calculations were run for the S=1/2 and S=5/2 spin states. None of the final optimized conformations displayed imaginary frequencies. The final reported energies correspond to the sum of electronic and thermal free energies.

Supplementary References

1. S. R. Marsden *et al.*, CH-π Interactions Promote the Conversion of Hydroxypyruvate in a Class II Pyruvate Aldolase. *Adv. Synth. Catal.* **361**, 2649-2658 (2019).
2. I. Salmeen, G. Palmer, Electron Paramagnetic Resonance of Beef-Heart Ferricytochrome c. *J. Chem. Phys.* **48**, 2049-2052 (1968).
3. A. Lundin, R. Aasa, A simple device to maintain temperatures in the range 4.2–100 K for EPR measurements. *J Magn. Res.* **8**, 70-73 (1972).
4. M. Cianci *et al.*, P13, the EMBL macromolecular crystallography beamline at the low-emittance PETRA III ring for high-and low-energy phasing with variable beam focusing. *J. Synchrotron Radiat.* **24**, 323-332 (2017).
5. W. Kabsch, Xds. *Acta Crystallogr. D* **66**, 125-132 (2010).
6. P. R. Evans, An introduction to data reduction: space-group determination, scaling and intensity statistics. *Acta Crystallogr. D* **67**, 282-292 (2011).
7. P. R. Evans, G. N. Murshudov, How good are my data and what is the resolution? *Acta Crystallogr. D* **69**, 1204-1214 (2013).
8. C. P. Collaborative, The CCP4 suite: programs for protein crystallography. *Acta Crystallogr. D* **50**, 760 (1994).
9. G. M. Sheldrick, A short history of SHELX. *Acta Crystallogr. A* **64**, 112-122 (2008).
10. T. Pape, T. R. Schneider, HKL2MAP: a graphical user interface for macromolecular phasing with SHELX programs. *J Appl. Crystallogr.* **37**, 843-844 (2004).
11. A. Vagin, A. Teplyakov, MOLREP: an automated program for molecular replacement. *J. Appl Crystallogr.* **30**, 1022-1025 (1997).
12. J. Painter, E. A. Merritt, A molecular viewer for the analysis of TLS rigid-body motion in macromolecules. *Acta Crystallogr. D* **61**, 465-471 (2005).
13. G. N. Murshudov *et al.*, REFMAC5 for the refinement of macromolecular crystal structures. *Acta Crystallogr. D* **67**, 355-367 (2011).
14. P. D. Adams *et al.*, PHENIX: a comprehensive Python-based system for macromolecular structure solution. *Acta Crystallogr. D* **66**, 213-221 (2010).
15. R. P. Joosten, F. Long, G. N. Murshudov, A. Perrakis, The PDB_REDO server for macromolecular structure model optimization. *IUCrJ* **1**, 213-220 (2014).
16. V. B. Chen *et al.*, MolProbity: all-atom structure validation for macromolecular crystallography. *Acta Crystallogr. D* **66**, 12-21 (2010).
17. J. Eberhardt, D. Santos-Martins, A. F. Tillack, S. Forli, *J. Chem. Inf. Model.* **61**, 3891-3898 (2021). DOI: 10.1021/acs.jcim.1c00203
18. C. E. Blanchet *et al.*, Versatile sample environments and automation for biological solution X-ray scattering experiments at the P12 beamline (PETRA III, DESY). *J Appl. Crystallogr.* **48**, 431-443 (2015).
19. M. A. Graewert *et al.*, Adding Size Exclusion Chromatography (SEC) and Light Scattering (LS) Devices to Obtain High-Quality Small Angle X-Ray Scattering (SAXS) Data. *Crystals* **10**, 975 (2020).
20. D. Franke, A. G. Kikhney, D. I. Svergun, Automated acquisition and analysis of small angle X-ray scattering data. *Nucl. Instrum. Meth. A* **689**, 52-59 (2012).
21. A. Panjkovich, D. I. Svergun, CHROMIXS: automatic and interactive analysis of chromatography-coupled small-angle X-ray scattering data. *Bioinformatics* **34**, 1944-1946 (2018).
22. A. Guinier (1939) La diffraction des rayons X aux trčs petits angles: application ą l'étude de phénomčnes ultramicroscopiques. in *Annales de physique*, pp 161-237.
23. K. Manalastas-Cantos *et al.*, ATSAS 3.0: expanded functionality and new tools for small-angle scattering data analysis. *J. Appl. Crystallogr.* **54** (2021).
24. P. V. Konarev, D. I. Svergun, A posteriori determination of the useful data range for small-angle scattering experiments on dilute monodisperse systems. *IUCrJ* **2**, 352-360 (2015).

25. D. Svergun, Determination of the regularization parameter in indirect-transform methods using perceptual criteria. *J. Appl. Crystallogr.***25**, 495-503 (1992).
26. N. R. Hajizadeh, D. Franke, C. M. Jeffries, D. I. Svergun, Consensus Bayesian assessment of protein molecular mass from solution X-ray scattering data. *Scientific reports* **8**, 1-13 (2018).
27. M. V. Petoukhov, D. I. Svergun, Ambiguity assessment of small-angle scattering curves from monodisperse systems. *Acta Crystallogr. D* **71**, 1051-1058 (2015).
28. D. Franke, C. M. Jeffries, D. I. Svergun, Machine learning methods for X-ray scattering data analysis from biomacromolecular solutions. *Biophys. J.* **114**, 2485-2492 (2018).
29. D. I. Svergun, M. V. Petoukhov, M. H. Koch, Determination of domain structure of proteins from X-ray solution scattering. *Biophys. J.* **80**, 2946-2953 (2001).
30. D. Svergun, C. Barberato, M. H. Koch, CRYSOL–a program to evaluate X-ray solution scattering of biological macromolecules from atomic coordinates. *J Appl. Crystallogr.* **28**, 768-773 (1995).
31. D. Franke, C. M. Jeffries, D. I. Svergun, Correlation Map, a goodness-of-fit test for one-dimensional X-ray scattering spectra. *Nat. Methods* **12**, 419-422 (2015).
32. A. G. Kikhney, C. R. Borges, D. S. Molodenskiy, C. M. Jeffries, D. I. Svergun, SASBDB: Towards an automatically curated and validated repository for biological scattering data. *Prot. Sci.* **29**, 66-75 (2020).
33 E. Krieger, R. L. Dunbrack, R. W. Hooft, B. Krieger, in *Computational Drug Discovery and Design*, Springer, **2012**, pp. 405-421.
34 E. Krieger, G. Vriend, *Bioinformatics* **2014**, *30*, 2981-2982.
35 M. Frisch, G. Trucks, H. Schlegel, G. Scuseria, M. Robb, J. Cheeseman, G. Scalmani, V. Barone, B. Mennucci, G. Petersson, H. Nakatsuji, M. Caricato, Gaussian Inc. Wallingford, CT **2009**, 32, 5648-5652.
36 A. Austin, G. A. Petersson, M. J. Frisch, F. J. Dobek, G. Scalmani, K. Throssell, *J. Chem. Theory Comput*. **2012**, *8*, 4989-5007.
37 S. Grimme, J. Antony, S. Ehrlich, H. Krieg, *J. Chem. Phys.* **2010**, *132*, 154104.
